# Development and characterization of a novel TDP‐43 positron emission tomography tracer: [^18^F]JNJ‐TDP43‐1

**DOI:** 10.1002/alz.71675

**Published:** 2026-07-18

**Authors:** Chunfang A. Xia, Mani Salarian, Christopher J. Gartshore, Antonella Scaglione, Thomas Hayes, Shuanglong Liu, Hsiu‐Ming Tsai, Javier Echavarren, Jose Maria Cid, Alessandra Matzeu, A. Katrin Szardenings

**Affiliations:** ^1^ Discovery Technologies and Molecular Pharmacology Johnson & Johnson San Diego California USA; ^2^ Neuroscience Biomarkers Johnson & Johnson San Diego California USA; ^3^ Global Discovery Chemistry Johnson & Johnson San Diego California USA; ^4^ Global Discovery Chemistry Johnson & Johnson Toledo Spain; ^5^ Preclinical Sciences and Translational Safety Johnson & Johnson San Diego California USA

**Keywords:** [^18^F]JNJ‐TDP43‐1, amyotrophic lateral sclerosis, frontotemporal lobar degeneration, limbic‐predominant age‐related TDP‐43 encephalopathy, neurodegenerative disorders, positron emission tomography, TDP‐43

## Abstract

**INTRODUCTION:**

Neurodegenerative disorders, including amyotrophic lateral sclerosis (ALS), frontotemporal dementia (FTD), limbic‐predominant age‐related TDP‐43 encephalopathy (LATE), and Alzheimer's disease (AD) are associated with TAR DNA‐binding protein 43 (TDP‐43) pathology. A positron emission tomography (PET) tracer targeting TDP‐43 aggregates could improve early diagnosis and guide treatment development for TDP‐43–related conditions.

**METHODS:**

Specific binding was evaluated using fluorescent labeling of compound, surface plasmon resonance (SPR), and autoradiography (ARG). Brain PET imaging in rats, nonhuman primate (NHP), and a disease mouse model was performed to characterize tracer pharmacokinetics and in vivo target binding.

**RESULTS:**

JNJ‐TDP43‐1 exhibited high binding affinity for pathological TDP‐43 (K_d_ = 7.1 nM) and remarkable selectivity over other proteinopathies. PET imaging demonstrated robust brain uptake and rapid washout in rodents and NHP. In vivo target engagement was confirmed in an AAV‐hTDP43 disease model.

**DISCUSSION:**

[^18^F]JNJ‐TDP43‐1 is a promising PET ligand for early diagnosis and evaluating therapies in TDP‐43‐related diseases.

## Highlights

- A novel positron emission tomography (PET) tracer selectively targets pathological TAR DNA‐binding protein 43 (TDP‐43).
- High affinity and potency: K_d_ = 7.1 nM measured by surface plasmon resonance (SPR) and IC_50_ = 10 nM detected by autoradiography (ARG).
- High selectivity over beta amyloid (Aβ), tau, and alpha‐synuclein (α‐syn) aggregates.
- Robust target engagement demonstrated in human and animal tissues by ARG.
- Favorable In vivo profile: good brain uptake and rapid washout in rats and nonhuman primate (NHP) PET studies.
- In vivo target binding: increased tracer retention at AAV‐driven phospho‐TDP‐43 overexpression site in mouse brains.

## 1 BACKGROUND

Neurodegenerative disorders (NDs) are characterized by the common feature of pathological protein aggregation, with beta‐amyloid (Aβ), tau, and alpha‐synuclein (*α*‐syn) being among the most prevalent proteins involved in these aggregates.^1^, ^2^, ^3^ However, recent studies have increasingly highlighted the significance of aggregated TAR DNA‐binding protein 43 (TDP‐43) as a critical component associated with a range of NDs.^4^ Amyotrophic lateral sclerosis (ALS) is a fatal, progressive neurodegenerative disease that affects motor neurons in the brain. In 97% of ALS patients, TDP‐43 proteinopathy is observed in the motor cortex and spinal cord.^5^ TDP‐43 proteinopathy is also reported *post mortem* in about 50% of frontotemporal dementia (FTD) cases, which are characterized as frontotemporal lobar degeneration with TDP‐43 pathology (FTLD‐TDP).^6^, ^7^ Limbic‐predominant age‐related TDP‐43 encephalopathy (LATE) was a recently recognized condition in older adults characterized by a stereotypical TDP‐43 proteinopathy that can coexist with or without amyloid plaques and Tau neurofibrillary tangles (Alzheimer's disease neuropathological change, ADNC).^8^ Additionally, TDP‐43 pathology is present in up to 57% of Alzheimer's disease (AD) cases, commonly appearing in the limbic system and linked to greater cognitive decline and memory loss.^9^, ^10^ TDP‐43 proteinopathies feature the accumulation of insoluble, misfolded TDP‐43 in the central nervous system (CNS), which occurs alongside progressive neuronal loss and gliosis.^11^ The inclusion of TDP‐43 aggregates is a pathological hallmark that contributes to the neurodegeneration observed in these conditions.^12^

Currently, TDP‐43 proteinopathy remains a *post mortem* pathologic diagnosis. Despite the increasing recognition of TDP‐43's role in neurodegenerative diseases, there is still a paucity of in vivo biomarkers and imaging techniques for evaluating TDP‐43 pathology. To date, the detection of TDP‐43 in biofluids (plasma, serum, and cerebrospinal fluid) has not been very successful.^13^ Direct measurement of TDP‐43 aggregates using positron emission tomography (PET) provides a powerful approach to improve diagnostic accuracy and to more effectively evaluate therapeutic responses in clinical trials. PET radiotracers targeting pathological *αβ* and tau have been successfully developed,^14^ offering unprecedented insight into the progression of molecular pathology in the living brain and its relationship to evolving clinical symptoms. Additionally, considerable efforts have also been directed toward the development of *α*‐syn tracers.^15^ While substantial progress has been achieved in detecting Aβ, tau, and *α*‐syn, no imaging biomarker for TDP‐43 have been established.

In the absence of biomarkers for TDP‐43, the diagnosis remains primarily based on clinical assessment and neuroimaging techniques that do not specifically target TDP‐43. One study attempted to utilize tau PET radioligands to image TDP‐43 in ALS and found that none of the tau ligands ([^3^H]MK‐6240, [^3^H]JNJ‐067, [^3^H]GTP‐1, [^3^H]CBD‐2115, [^3^H]Flortaucipir, or [^3^H]APN‐1607) are suitable for selective imaging of TDP‐43 in ALS for clinical research purposes.^16^ Another study indicated that fluorodeoxyglucose (FDG) PET may be utilized for detecting AD related TDP‐43 proteinopathy, which is associated with hypometabolism in the medial temporal and frontal brain regions.^17^ Last year, AC Immune published their discovery of the first TDP‐43 PET tracer [^18^F]ACI‐19626 with the binding affinity (K_d_ = 24 and 25 nM) to FTLD‐TDP type A and type B TDP‐43 aggregates, respectively, which represents an important milestone toward imaging TDP‐43 pathology in vivo.^18^ Overall, the identification of potent and selective radiotracers for pathological TDP‐43 remains an ongoing challenge.

Our goal was to develop a PET imaging ligand with high affinity and selectivity for TDP‐43 aggregates, along with a favorable in vivo profile. We evaluated the tracer using in vitro fluorescent labeling with compound, autoradiography (ARG), surface plasmon resonance (SPR) binding assays, and in vivo PET imaging. In this study, we present the synthesis and characterization of [^18^F]JNJ‐TDP43‐1, accompanied by detailed assessments of its binding properties and in vivo pharmacokinetic (PK) and binding profile. The radiotracer [^18^F]JNJ‐TDP43‐1 holds promise as a diagnostic tool for identifying, characterizing, and monitoring TDP‐43 pathology. Our findings contribute to the evolving field of neuroimaging and pave the way for future research aimed at elucidating the role of TDP‐43 in NDs.

## 2 METHODS

### 2.1 Human brain samples

*Post mortem* frozen tissue samples from different brain regions of healthy donors and donors with confirmed TDP‐43 or other (Aβ, tau, and *α*‐syn) pathologies were acquired from ABS Bio (Irvine, CA), the Anatomy Gifts Registry (Gaithersburg, MD), and Banner Health (Phoenix, AZ). Table S1 presents comprehensive data on human brain samples, including clinical diagnosis (Case ID), *post mortem* diagnosis, demographic details and neuropathological findings. Healthy controls (HC) ranged in age from 39 to 84 years (65 ± 21 years), whereas donors with *post mortem* diagnoses of ALS, ALS/FTD, FTLD‐TDP, LATE‐NC/ADNC, or Parkinson's disease (PD) ranged from 43 to 93 years (69 ± 16 years).

**RESEARCH IN CONTEXT**

1. **Systematic review**: TAR DNA‐binding protein 43 (TDP‐43) aggregation is a pathological hallmark of amyotrophic lateral sclerosis (ALS), frontotemporal lobar degeneration (FTLD), limbic‐predominant age‐related TDP‐43 encephalopathy (LATE), and as a co‐pathology in Alzheimer's disease (AD). Currently, no clinically established positron emission tomography (PET) tracer is available for in vivo imaging of TDP‐43 pathology, highlighting a critical unmet need for a selective, high‐affinity radioligand.
2. **Interpretation**: We report [^18^F]JNJ‐TDP43‐1 as a novel TDP‐43 PET ligand with high binding affinity (K_d_ = 7.1 nM) and potency (IC_50_ = 10 nM), together with strong selectivity over beta amyloid (Aβ), tau, and alpha synuclein(*α*‐syn) aggregates. In vivo PET studies in rats, nonhuman primates(NHP), and an AAV‐hTDP‐43 mouse model, the tracer demonstrated favorable brain penetration, rapid washout, and in vivo target engagement, minimal off‐target binding, and good metabolic stability.
3. **Future directions**: These findings support clinical translation of this TDP‐43 PET tracer for human imaging studies.

### 2.2 SPR assay

The SPR assay was used to measure the binding affinity and selectivity of the compound, as previously reported by B. Sun et al.^19^ All antibody information for the SPR assay is listed in Table S2. A Series S CM5‐coated SPR chip (Cytiva, CA) was immobilized with 30 µg/mL of the phosphorylated TDP‐43 (pTDP‐43) antibody using a Biacore S200 system (GE, IL). Cytoplasmic fractions from HC, ALS, ALS/FTD, FTLD‐TDP, and LATE‐NC/ADNC human brain samples were prepared according to the manufacturer's protocol using the Subcellular Protein Fractionation Kit (ThermoFisher, MA, Cat# 87790). The detailed procedure is provided in Materials S1. Cytoplasmic samples were diluted to 4 mg/mL in running buffer (10 mM HEPES, pH 7.4, 1.5 M NaCl, 0.03 M ethylenediaminetetraactic acid [EDTA], 0.5% Surfactant P20, and 5% dimethyl sulfoxide [DMSO]). Following capture of TDP‐43 aggregates on the pTDP‐43 antibody‐coated surface, JNJ‐TDP43‐1 was injected in a seven‐point, four‐fold serial dilution series ranging from 0.02 to 100 nM. The equilibrium dissociation constant (K_d_) was determined using the Biacore Evaluation Software with a 1:1 binding model.

A similar protocol was used to assess binding selectivity against Aβ, tau, and *α*‐syn. Antibodies against Aβ42, phosphorylated tau (p‐tau), and phosphorylated *α*‐syn (p‐syn) were immobilized on the SPR chip. Brain samples with pathological Aβ_42_, tau, and/or *α*‐syn (listed in Table S1) were homogenized in ice‐cold RIPA lysis and extraction buffer (ThermoScientific, IL, Cat# 89901) with protease/phosphatase inhibitors (ThermoScientific, IL) following incubation for 15–30 minutes on ice. Samples were then centrifuged at 13,000 × *g* at 4°C for 15 minutes. The supernatants were captured at equivalent concentrations (4 mg/mL) on their respective antibody‐coated surfaces. The selective binding of JNJ‐TDP43‐1 over Aβ42, p‐tau, and p‐syn was then evaluated using the same procedure described above.

### 2.3 Mouse model

All rodent experiments and procedures were conducted in accordance with the Institutional Animal Care and Use Committee (IACUC) protocols at Johnson & Johnson. A mouse model of TDP‐43 aggregates was generated by administration of an AAV9 carrying the h*TARDBP* gene with A315T and G348C mutations. In this model, 2 µL of AAV9‐hSYN1‐h*TARDBP*(A315T/G348C)‐WPRE (AAV‐hTDP43) or AAV9‐hSYN1‐Null‐WPRE (AAV‐Null) particles (3 × 10^13^ GC/mL) (Vector Biolabs, PA) were delivered to the left cortex or substantia nigra (SN) of wild‐type (WT) C57BL/6J female mice (12 weeks old, Jackson Laboratory, ME, Strain #000664) via intracranial injections. Injection coordinates for the left cortex were anteroposterior (AP), +2.2 mm; lateral, +1.7 mm; and dorsoventral (DV), −3.0 mm from bregma.^20^ For the left SN, coordinates were AP, −3.0 mm; mediolateral, −1.3 mm; and DV, −4.2 mm from dura.^21^ A micro syringe injector and controller (Stoelting, IL) delivered 2 µL of viral particles. Mice injected into the left cortex (*n* = 3 for AAV‐hTDP43, *n* = 2 for AAV‐Null) underwent PET imaging 4 weeks after injection. Subsequently, brain samples were collected for immunohistochemistry (IHC) analysis to correlate with the PET signal.

### 2.4 Immunohistochemistry

Tissue sections were prepared from fresh frozen human brain blocks and mouse brains injected with AAV‐hTDP43 or AAV‐Null. The fresh frozen sections (10 µm) were air‐dried and fixed with Paraformaldehyde Fixative Solution (Alfa Aesar, MA). Mouse samples were quenched with 3% H_2_O_2_ for 30 minutes. While human samples were treated with 0.25% KMnO4 in phosphate buffered saline (PBS) for 12 minutes and followed by neutralizing using 0.1% potassium metabisulfite and 0.1% oxalic acid solution. After blocking with 10% goat serum, the sections were incubated overnight with primary antibody (see Table S2). A secondary antibody (Invitrogen, CA) was then applied for visualization, and images were captured using a Zeiss Axio Imager M2 (Zeiss, Germany). The bright‐field staining was performed with a Leica Bond Rx autostainer (Leica, IL), followed by imaging on a Vectra PolarisTM Quantitative Pathology Imaging System (Akoya Biosciences, MA).^22^ Images were analyzed by HALO link 4.2 (Indica labs, NM). To assess target engagement, IHC and ARG assays were performed on adjacent sections for side‐by‐side comparison.

### 2.5 Fluorescent staining with compound

JNJ‐TDP43‐1 exhibited weak autofluorescence and was evaluated through fluorescent staining on tissue sections, followed by pTDP‐43 IHC to determine its specific binding to pathological TDP‐43. Fresh frozen sections of human brains obtained from healthy donors as well as individuals with FTLD‐TDP, ALS, LATE‐NC/ADNC, and PD were quenched after fixation. The sections were incubated with 100 mM of JNJ‐TDP43‐1 in PBS for 1 h at room temperature. Following fluorescent staining with compound, IHC was performed using antibodies against pTDP‐43, p‐tau, Aβ_42_, and p‐syn as described previously, and the colocalization of compound and antibody signals was then assessed.

### 2.6 Radiosynthesis of [^18^F]JNJ‐TDP43‐1

The [^18^F]JNJ‐TDP43‐1 precursor (JNJ‐TDP43‐2) and standard were prepared in two steps. Detailed synthesis and radiosynthesis of JNJ‐TDP43‐1 as well as analytical data for each intermediate, precursor and standard are provided in detail in Materials S2 and S3. Briefly, the [^18^F]JNJ‐TDP43‐1 tracer was prepared on an automated synthesis module. A solution of the precursor in DMSO was heated with anhydrous [^18^F]fluoride at 180°C to yield the desired tracer (Figure S1). After high‐performance liquid chromatography (HPLC) purification, the [^18^F]JNJ‐TDP43‐1 was retained on a C18 SepPak Light cartridge and eluted with 10% ethanol/ 90% saline solution.

### 2.7 [^18^F]JNJ‐TDP43‐1 ARG

Each air‐dried frozen section was incubated with 200 µCi/mL of [^18^F]JNJ‐TDP43‐1 at room temperature for 60 minutes in incubation buffer (2.5% DMSO + 2.5% ethanol + 95% PBS). To determine non‐specific binding (NSB), adjacent brain sections were incubated with [^18^F]JNJ‐TDP43‐1 mixed with 10 µM non‐radiolabeled JNJ‐TDP43‐1. The sections were then subjected to a series of washes: 2 minutes in PBS, 1 minute in 30% ethanol/PBS, 1 minute in 50% ethanol/PBS, and 2 minutes in PBS to remove unbound tracer, followed by two dips in cold water. After drying, the labeled sections were exposed to a phosphor screen (Cytiva, MA) overnight. Autoradiographic images were then obtained from a Typhoon biomolecular imager (Cytiva, MA) and analyzed using ImageQuantTL (Cytiva, Marlborough, MA). Specific binding (SB) was calculated by subtracting NSB from total binding (TB) ARG signal. The percentage of blocking was then determined as % Blocking = (SB / TB) × 100, reflecting the proportion of total signal that is displaceable.

To determine the IC_50_ of JNJ‐TDP43‐1, ARG was performed on nine adjacent human motor cortex sections from ALS brain. [^18^F]JNJ‐TDP43‐1 was co‐incubated with varying concentrations of non‐radiolabeled JNJ‐TDP43‐1 (0, 0.001, 0.003, 0.01, 0.03, 0.1, 0.3, 1, and 3 µM). The percentage of inhibition was subsequently calculated.

### 2.8 PET imaging studies in mice and rats

Mice were anesthetized using isoflurane (2.0%–3.5% for induction and 1.0%–2.5% for maintenance) in air. [^18^F]JNJ‐TDP43‐1 (∼5.5 MBq) was administered in 5% ethanol in saline by tail vein injection. A 30‐minute dynamic PET scan was performed on a GNEXT PET/CT scanner (Xodus Imaging, Culver City, CA) for data acquisition, followed by a 1‐minute standard CT. Both CT and PET data were reconstructed using the system acquisition software. CT data were reconstructed using an ordered‐subsets expectation maximization (OSEM) algorithm with a 200 µm isotropic voxel size for anatomical co‐registration and attenuation correction. PET images were reconstructed using an iterative three‐dimensional (3D) OSEM algorithm with attenuation and decay correction. The data were reconstructed into 256 × 256 matrix images. PMOD software v4.2 (PMOD Technologies, Switzerland) was used for data analysis, quantification of time‐activity curves (TACs), assessment of overall brain uptake of the PET radioligand, and calculation of standardized uptake values (SUVs), as shown below:

$$SUV\;\left[\frac{g}{mL}\right]\;\left(BW\right)=\frac{C\left(PET\right)\left(T\right)\left[\frac{\mathrm{kBq}/\mathrm{mL}}{mL}\right]}{\frac{Injected\;Dose\;\left[\mathrm{kBq}\right]}{Bodyweight\;\left[g\right]}}$$

PMOD‐PNROD was used for mouse brain Atlas data analysis. PET and CT images were re‐orientated for affine matching of the brain atlas orientation. For SUV ratio (SUVR) measurements, cerebellum was used as a reference region.

For rat imaging, normal male Sprague–Dawley rats (8–10 weeks old, Charles River Laboratories, CA, *n* = 2) were placed on the imaging bed of a Siemens Inveon microPET/CT MM scanner (Siemens, Knoxville). The imaging protocol started with a CT scan followed by a dynamic PET scan (0–60 minutes) immediately after intravenous injection of ∼14 MBq of [^18^F]JNJ‐TDP43‐1 in 300 µL saline into each rat under 2% isoflurane in oxygen anesthesia. The upper body region was centered in the axial field of view of the scanner (103 mm axial length, two bed positions) to maximize sensitivity and resolution. CT acquisition was conducted at 80 kV and 500 µA. The CT scan was used for attenuation correction, anatomical images, and scatter correction for PET images. The PET scan energy window was set between 350 and 650 keV, with a 3.438 ns timing window. Emission data was collected in list mode for 60 minutes. PET images were reconstructed using a 3D‐OSEM reconstruction algorithm.

### 2.9 PET imaging studies in NHPs

The NHP study was performed by Perceptive Inc. (New Haven, CT, USA) at Yale University in full compliance with the University's IACUC policies and procedures. One female rhesus macaque (Macaca mulatta, 13 years old) from Yale's existing in‐house colony was used as a research animal and was returned to the colony at the end of the study. The rhesus macaque (6.1 kg) was injected with 175.2 MBq of [^18^F]JNJ‐TDP43‐1 and a dynamic PET scan was performed over 240 minutes. Brain imaging data were acquired on the microPET Focus 220 scanner (Siemens, Knoxville, TN). Following intravenous injection of the radioligand, dynamic emission data were collected continuously in list mode for 240 minutes and subsequently rebinned into a series of 57 dynamic 3D PET image volumes as follows: 6 × 0.5 minutes, 3 × 1 minutes, 2 × 2 minutes, and 46 × 5 minutes. A transmission scan was performed to provide correction coefficients for photon attenuation in matter. The dynamic series were subsequently reconstructed using filtered back projection with standard corrections for detector normalization, deadtime, randoms, scatter, attenuation, and radioactive decay provided by the camera manufacturer. A single 3.0 T T1 magnetic resonance imaging (MRI) of the brain was acquired prior to the PET study for anatomical reference and image processing. Reconstructed dynamic brain PET images were transferred and analyzed using the PMOD software package, v3.802. The PET images were rigidly aligned to the previously acquired animal's brain T1 MRI. The animal's brain MR image was spatially normalized to a common rhesus brain MR template, where brain volumes of interest (VOIs) were defined. The atlas was applied to each dynamic image to extract the average activity concentration (kBq/cc) within each VOI and generate TACs representing regional brain activity concentration over time. TACs were expressed as SUVs (g/mL), normalized to animal weight and injected dose.

### 2.10 Statistical analysis

The statistical analysis was conducted using GraphPad Prism 9 software (Prism, CA). Data are shown as the mean ± standard deviation (SD). An ordinary one‐way analysis of variance (ANOVA) with multiple comparisons was employed to compare three or more groups, while comparisons between two groups were performed using a t‐test. *p*‐Value < 0.05 indicates statistical significance.

## 3 RESULTS

### 3.1 Synthesis and radiosynthesis of JNJ‐TDP43‐1

**FIGURE 1:**
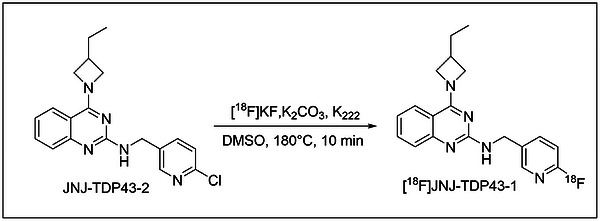
Radiosynthesis of [^18^F]JNJ‐TDP43‐1.

The structure of the tracer [^18^F]JNJ‐TDP43‐1 and its precursor, JNJ‐TDP43‐2, as well as the radiolabeling reaction, are shown in Figure 1. The average radiochemical yield for [^18^F]JNJ‐TDP43‐1 was 28 ± 7% (*n* = 9, decay‐corrected yield), with a radiochemical purity of > 95% and a molar activity of 52 ± 14 GBq/µmol (*n* = 9).

### 3.2 Binding affinity and selectivity of JNJ‐TDP43‐1 evaluated by SPR assay

**FIGURE 2:**
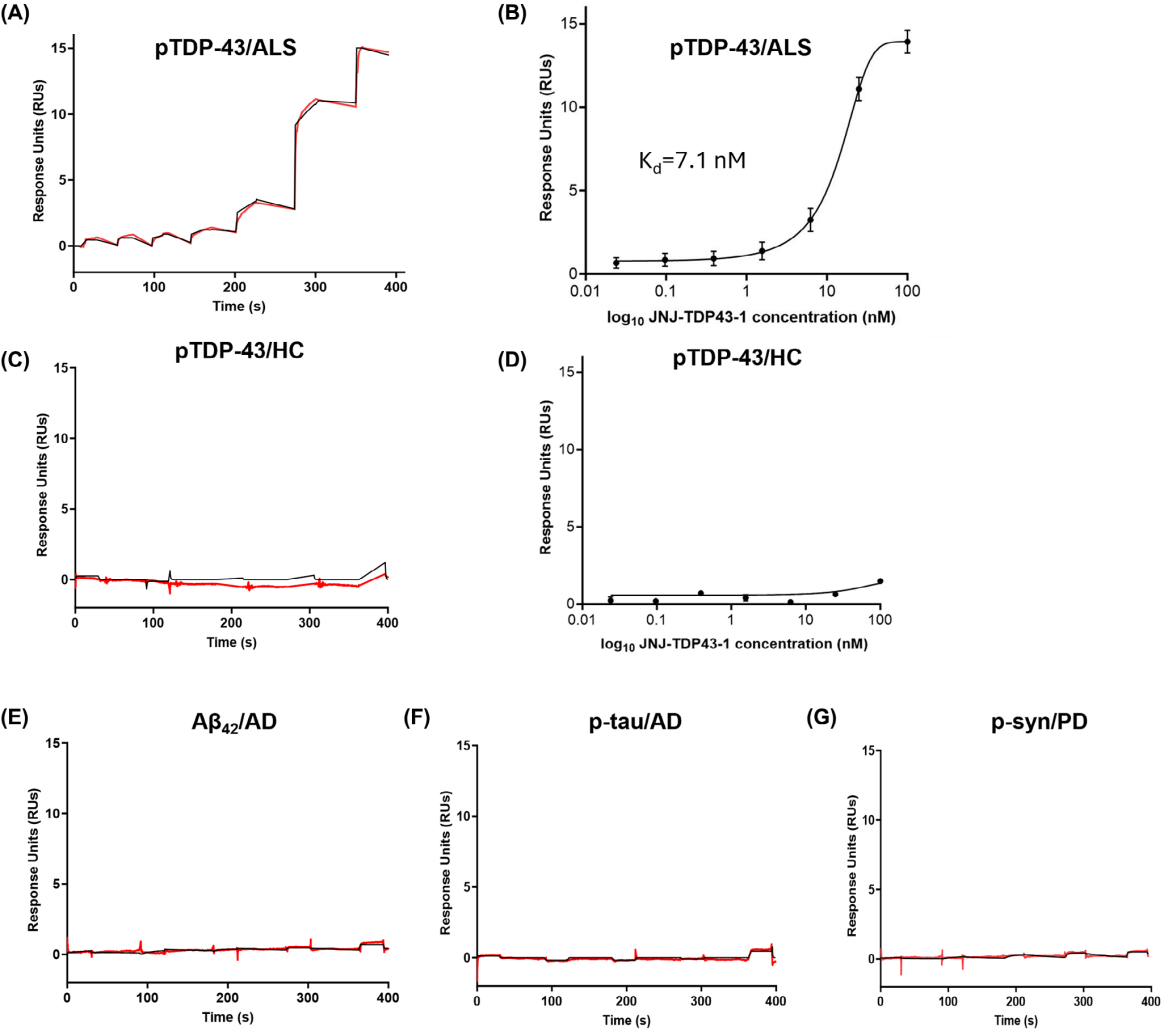
SPR‐based determination of the binding affinity and selectivity of JNJ‐TDP43‐1. Single‐cycle kinetic SPR analyses were performed to assess the interaction of JNJ‐TDP43‐1 with pathological TDP‐43 and to evaluate selectivity against other neurodegeneration‐associated aggregated proteins. (A) Representative SPR sensorgram showing JNJ‐TDP43‐1 binding to pTDP‐43 derived from an ALS brain cytoplasmic sample. (B) Corresponding fitted binding curves for K_d_ measurement obtained across 15 human FTLD‐TDP, ALS, ALS/FTD, and LATE‐NC/ADNC cytoplasmic samples (mean ± SD), demonstrating consistent high‐affinity interaction with pathological TDP‐43 species. (C–D) No measurable binding of JNJ‐TDP43‐1 was detected in cytoplasmic fractions from healthy control brain samples. (E–G) Representative SPR sensorgrams illustrating the lack of binding to pathological Aβ, tau, or *α*‐syn captured from AD or PD brain homogenates, confirming target selectivity. In all SPR sensorgrams, red line = experimental curve, black line = fitting curve.

Western blot (WB) analysis was conducted to assess pTDP‐43 levels in brain homogenates. The results confirmed the presence of pathological TDP‐43 inclusions within the cytoplasm. Additionally, increased levels of pathological TDP‐43 were observed in disease cohorts (ALS, ALS/FTD, FTLD‐TDP, LATE‐NC/ADNC), whereas control samples exhibited only minimal levels. WB methodology is detailed in Materials S4, and representative WB images are provided in Figure S2. The binding affinity of JNJ‐TDP43‐1 for pathological TDP‐43 was assessed using cytoplasmic fractions prepared from human ALS (*n* = 4), ALS/FTD (*n* = 2), FTLD‐TDP (*n* = 6), and LATE‐NC/ADNC (*n* = 3) brain samples, with healthy donor brain included as a control (*n* = 4). SPR single‐cycle kinetics sensorgrams and corresponding fitted binding curves are shown in Figures 2a–d. JNJ‐TDP43‐1 exhibited a clear concentration‐dependent affinity for pathological TDP‐43 aggregates (Figure 2a,b), resulting in an overall mean K_d_ of 7.1 ± 2.5 nM (*n* = 15, Table 1). The dissociation constants K_d_ varied across disease types as follows: FTLD‐TDP, 7.6 ± 2.3 nM (*n* = 6); ALS, 8.4 ± 1.3 nM (*n* = 4); ALS/FTD, 8.0 ± 1.4 nM (*n* = 2); and LATE‐NC/ADNC, 3.9 ± 2.8 nM (*n* = 3). In contrast, no measurable binding was detected in healthy control brain samples (K_d_ > 500 µM, Figure 2c,d), confirming the compound's selectivity for pathological TDP‐43 species.

**TABLE 1:** Affinity K_d_ determination of JNJ‐TDP43‐1 by SPR assay.

Antibody	pTDP‐43					Aβ_42_	p‐tau	p‐syn
Brain lysate	HC	FTLD‐TDP	ALS	ALS/FTD	LATE‐NC/ADNC	LATE‐NC/ADNC	LATE‐NC/ADNC	PD
Mean K_d_ (nM)	569000	7.6	8.4	8.0	3.9	18900	8290	20700

*Note*: The average K_d_ to pTDP‐43 was determined using 15 human brain samples (including FTLD‐TDP, ALS, ALS/FTD and LATE‐NC/ADNC), K_d_ = 7.1 ± 2.5 nM. The selectivity assay (K_d_) was conducted on LATE‐NC/ADNC brain homogenates for Aβ_42_ and p‐tau, as well as on PD brain samples for p‐syn measurements.

In addition, SPR assays were used to evaluate the binding selectivity of JNJ‐TDP43‐1 for pathological Aβ, tau, and *α*‐syn in brain samples from patients with ADNC (for Aβ_42_ and p‐tau) or PD (for p‐syn). The sensorgrams shown in Figure 2e–g display only minimal and nonprogressive signal changes. JNJ‐6067^23^ and Pittsburgh Compound B (PIB)^24^ were used as positive controls. JNJ‐6067 demonstrated a K_d_ of 0.96 nM for p‐tau, while PIB had a K_d_ of 3.5 nM for Aβ_42_, measured in LATE‐NC/ADNC brain samples captured with anti‐p‐tau and anti‐Aβ_42_ antibodies, respectively. A positive control for PD was omitted because of the absence of a suitable *α*‐syn binder. These profiles do not resemble specific association–dissociation kinetics and are therefore consistent with nonspecific interactions. Global sensorgram fitting failed to yield meaningful binding parameters for Aβ42 (K_d_ = 18900 nM), p‐tau (K_d_ = 8290 nM), or p‐syn (K_d_ = 20700 nM) aggregates, indicating that JNJ‐TDP43‐1 lacks significant specific binding to these targets under the tested conditions shown in Table 1.

### 3.3 Fluorescent compound staining of JNJ‐TDP43‐1

**FIGURE 3:**
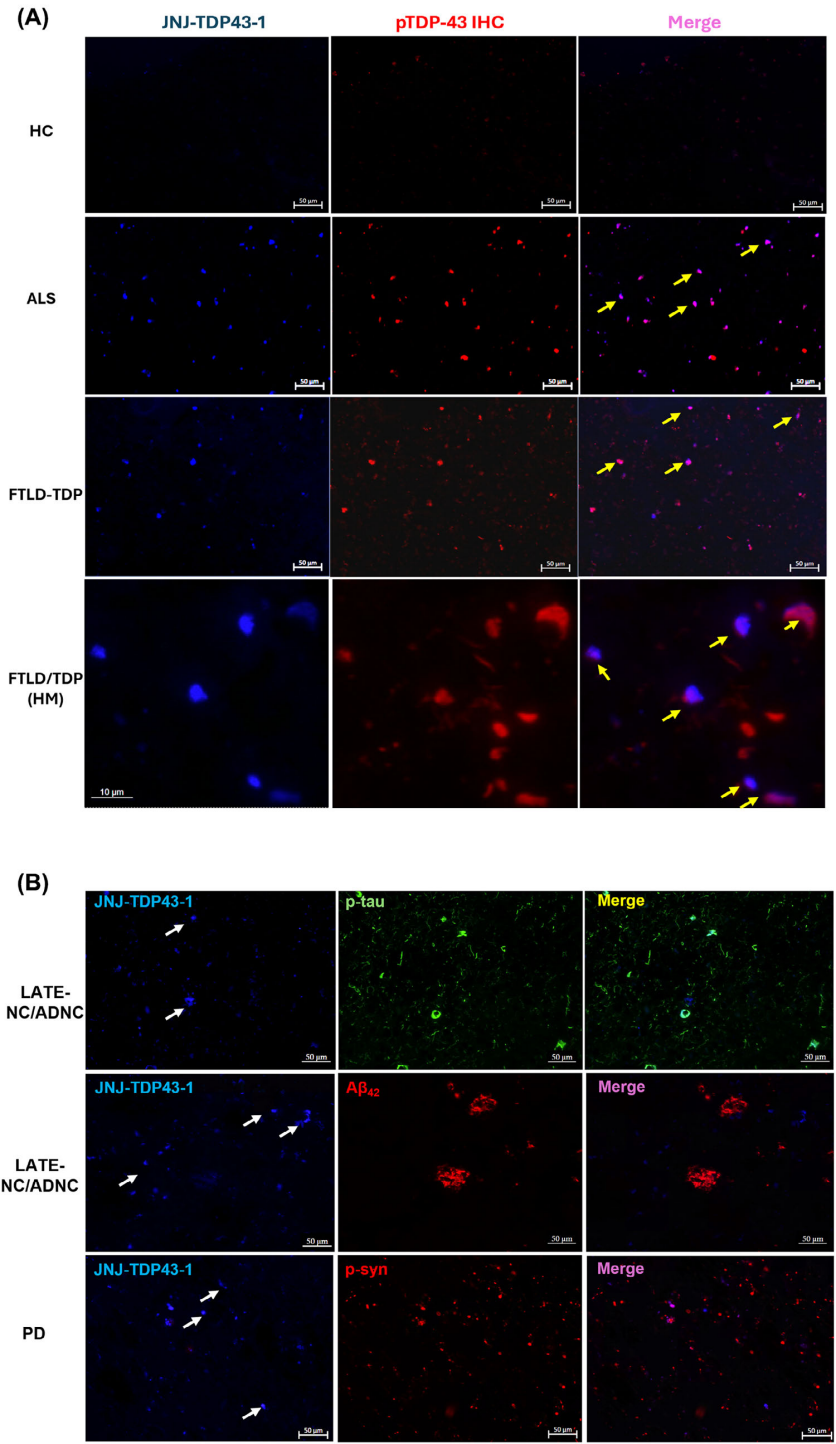
Fluorescence staining of JNJ‐TDP43‐1 on human *post mortem* brain sections across neurodegenerative proteinopathies. (A) Representative fluorescence microscopy images of JNJ‐TDP43‐1 (blue) and pTDP‐43 IHC (red) in healthy control (HC), ALS, and FTLD‐TDP brain sections. Merged images demonstrate colocalization of JNJ‐TDP43‐1 fluorescent staining with pTDP‐43‐positive inclusions in ALS and FTLD‐TDP cases. Yellow arrows indicate representative TDP‐43‐positive pathological inclusions. HM = high magnification. Scale bar = 50 uM or 10 µm in HM images. Minimal signal was observed in HC tissue, confirming specificity of binding. (B) JNJ‐TDP43‐1 selectivity was tested against non‐TDP‐43 proteinopathies by comparing its fluorescence (blue) with IHC for p‐tau (green), Aβ_42_ (red) in LATE‐NC/ADNC brain sections, and p‐syn (red) in Parkinson's disease. Merged images were used to assess colocalization, and white arrows highlight isolated JNJ‐TDP43‐1 staining that does not overlap with the other pathologies. JNJ‐TDP43‐1 showed selective labeling of TDP‐43 aggregates without appreciable cross‐binding to pathological tau, Aβ, or *α*‐syn deposits. Scale bars = 50 µm.

JNJ‐TDP43‐1 solution exhibited autofluorescence on the DAPI (4′,6‐diamidino‐2‐phenylindole) channel, permitting visualization by fluorescence microscopy. However, the signal was relatively weak, and a higher concentration (100 mM) was required for reliable detection. On ALS motor cortex and FTLD‐TDP temporal cortex sections, compound staining showed positive binding that colocalized with pTDP‐43 IHC, as indicated by the yellow arrows in Figure 3a. The compound labeled only a subset of pTDP‐43–positive structures, likely reflecting differences in aggregate accessibility between antibody detection and small molecule binding. Compound's weak autofluorescence may also lead to underestimation of actual target binding. On HC brain sections, only minimal positive staining of JNJ‐TDP43‐1 was seen, and no positive immunoreactivity was detected by IHC staining on the same section.

Figure 3b shows that JNJ‐TDP43‐1 selectively labels TDP‐43 aggregates, with little to no binding to pathological tau, Aβ, or *α*‐syn. Double fluorescence staining with JNJ‐TDP43‐1 and IHC was performed on LATE‐NC/ADNC brain sections for p‐tau (green) and Aβ_42_ (red), and on PD sections for p‐syn (red). White arrows indicate the absence of colocalization between JNJ‐TDP43‐1 and these pathologies, supporting selective labeling of TDP‐43 aggregates with minimal cross‐reactivity. In PD tissue, minimal overlap with *α*‐syn was observed, possibly due to nonspecific or off‐target binding in areas of dense protein aggregation; however, the overall fluorescence pattern remained distinct from *α*‐syn pathology.

### 3.4 ARG and IHC comparison on adjacent sections

**FIGURE 4:**
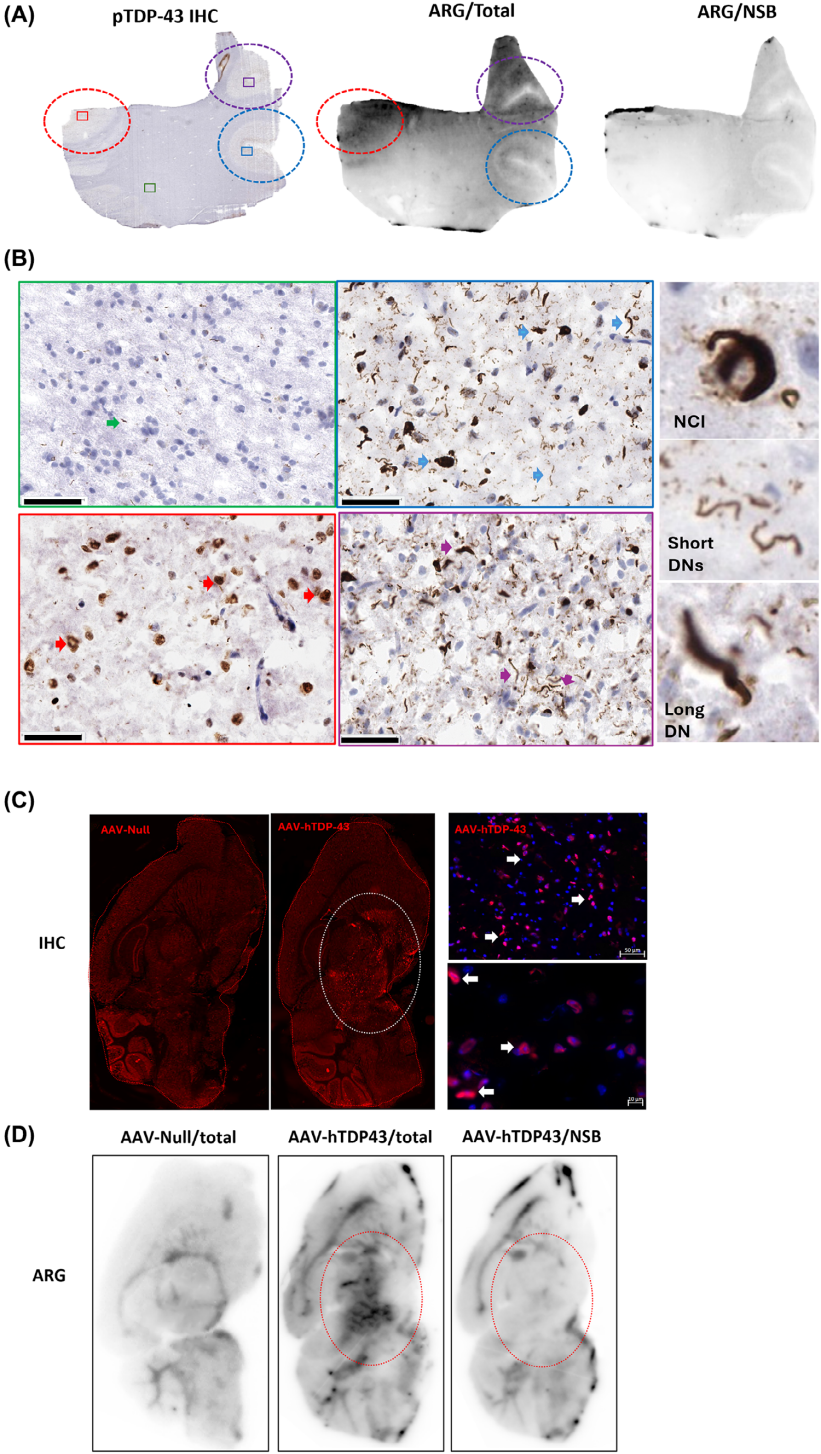
Comparison of [^18^F]JNJ‐TDP43‐1 ARG and pTDP‐43 IHC on adjacent brain sections. (A) Adjacent human FTLD‐TDP temporal cortex sections were stained with pTDP‐43 IHC or [^18^F]JNJ‐TDP43‐1 ARG. Whole‐section images display IHC (left), ARG total binding (center), and ARG NSB (right). Colored dashed circles on the IHC section mark regions of interest that match positions on the ARG images. Small colored rectangles on the IHC panel show source fields for higher‐magnification views. (B) Colored rectangles show pathological TDP‐43 deposits in different regions at higher magnification. Scale bar = 50 µm. The insets on the right show TDP‐43 pathology types: neuronal cytoplasmic inclusions (NCI), short dystrophic neurites (short DNs), and long dystrophic neurites (long DNs). (C) Fluorescent IHC of pTDP‐43 on mouse brain sagittal sections collected 4 weeks postinjection of the AAV‐Null (left) or AAV‐hTDP43 (middle) vectors. Higher‐magnification images on the right show human pTDP‐43 overexpression in mouse brain after AAV‐hTDP43 administration, with arrows marking cytoplasmic inclusions. (D) ARG was performed on adjacent sections. Red ovals highlight corresponding regions where hTDP43 overexpression was identified by IHC in AAV‐hTDP43‐injected brain tissue.

To evaluate the tracer's in vitro target engagement, total and NSB ARG of [^18^F]JNJ‐TDP43‐1, along with pTDP‐43 IHC, were performed on adjacent brain sections from FTLD‐TDP temporal cortex (Figure 4a,b) as well as whole mouse brains (Figure 4c,d) administered with AAV‐Null or AAV‐hTDP43 vectors. In human FTLD‐TDP brain sections, colored circles denote regions exhibiting increased total ARG signal, which align with areas abundant in pTDP‐43 aggregates, as illustrated in Figure 4a. Colored rectangles delineate specific regions that either contain or lack pathological TDP‐43 deposits (Figure 4b). Red, blue, and purple regions exhibited high levels of pTDP‐43 inclusions, whereas the green region (white matter) showed very low pTDP‐43 expression, corresponding to areas with reduced ARG signal. The insets on the right display TDP‐43 neuronal cytoplasmic inclusions (NCI), short dystrophic neurites (short DNs), and long dystrophic neurites (long DNs). The positive ARG signals observed across all regions were effectively displaced by 10 µM JNJ‐TDP43‐1, demonstrating the tracer's specific binding to pathological TDP‐43 in human brain tissue.

Fluorescent IHC of pTDP‐43 was also conducted on mouse sagittal brain sections collected 4 weeks after SN injection of the AAV‐Null (left) or AAV‐hTDP43 (middle) vectors (Figure 4c). Following AAV‐hTDP43 administration, elevated human pTDP‐43 levels appeared near the SN region (white oval). At higher magnification, cytoplasmic TDP‐43 inclusions were observed, indicated by white arrows. Non‐specific IHC staining was observed in the cerebellum after both AAV‐Null and AAV‐hTDP43 injections, likely due to its dense structure and numerous epitopes that often cause such reactivity of antibody. The increased pTDP‐43 expression corresponded to the total ARG signal observed in the adjacent section (red oval, Figure 4d). This ARG signal was effectively displaced by co‐incubation with 10 µM non‐radiolabeled JNJ‐TDP43‐1 (NSB), further confirming the tracer's specific binding to pathological TDP‐43. Minimal signal in the AAV‐Null section for both IHC and ARG confirms that [^18^F]JNJ‐TDP43‐1 binding is specific to TDP‐43 pathology driven by the AAV construct rather than endogenous background. A low level of non‐specific signal was also noted in white matter regions, which was not reduced by the cold compound, indicating residual non‐displaceable binding.

### 3.5 ARG on different human brain sections and IC_50_ measurements

**FIGURE 5:**
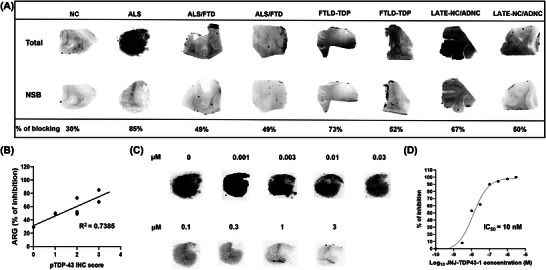
Specific binding of [^18^F]JNJ‐TDP43‐1 to TDP‐43 aggregates and IC_50_ measured by in vitro ARG. (A) [^18^F]JNJ‐TDP43‐1 ARG on different human brain sections. Total = total binding; NSB = non‐specific binding with co‐incubation of 10 µM JNJ‐TDP43‐1. (B) The correlation between the pTDP‐43 IHC score (see Table S1) and ARG signal (% of blocking). (C–D) The concentration‐dependent inhibition of [^18^F]JNJ‐TDP43‐1 binding is shown in the inhibition curve, yielding an IC_50_ value of 10 nM.

ARG was performed on human brain sections obtained from eight donors, which included normal control (NC, frontal cortex), ALS (motor cortex), 2× ALS/FTD (motor cortex), 2× FTLD‐TDP (temporal cortex), and 2× LATE‐NC/ADNC (middle frontal gyrus of frontal cortex), see Figure 5a. Strong ARG signal was observed on sections from ALS, ALS/FTD, FTLD‐TDP, as well as LATE‐NC/ADNC brains. The lowest ARG signal was observed on normal brain sections. The blocking effect was found on normal human brain (∼30%) and 49%–85% of self‐blocking effects on disease brain sections. The highest blocking effect was found on ALS brain sections. The correlation between the pTDP‐43 IHC score (Table S1) and ARG signal (% of blocking) was measured. Two factors demonstrated positive correlation and *R^2^* = 0.7385. *P* value = 0.0062 (Figure 5b).

To determine the IC_50_ of JNJ‑TDP43‑1, motor cortex sections from ALS brain tissue were selected due to the high levels of TDP‐43 pathology identified by both IHC and ARG. In vitro ARG was then performed using the [^1^ ^8^F]JNJ‑TDP43‑1 tracer (Figure 5c). The assay showed that JNJ‐TDP43‐1 exhibits an IC_50_ of 10 nM (Figure 5d), closely matching the Kd value (7.1 nM) measured by the SPR assay (Table 1).

### 3.6 Assessment of the in vivo PK of [^18^F]JNJ‐TDP43‐1 in WT rats and rhesus macaques

**FIGURE 6:**
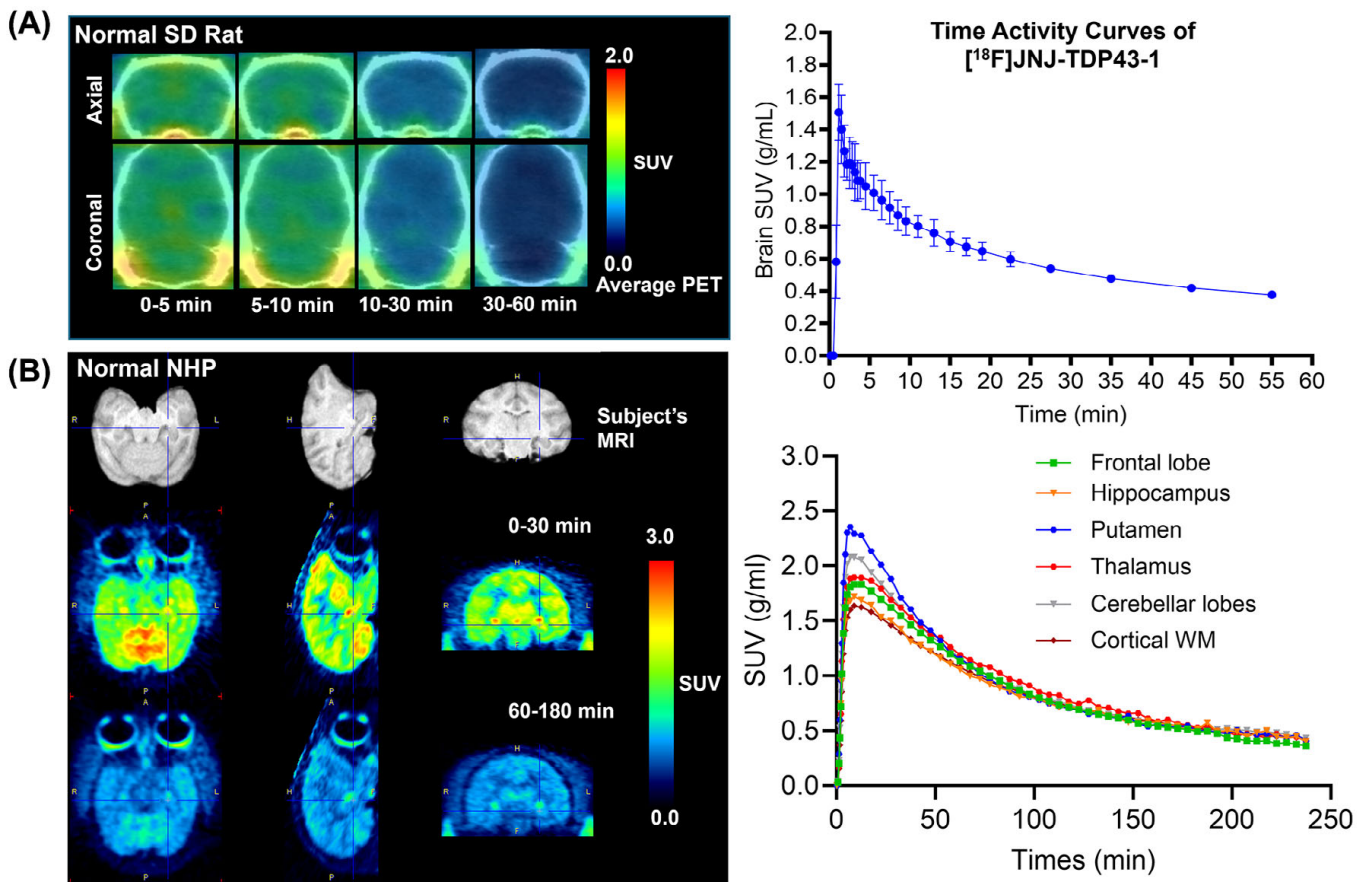
Brain PK of [^18^F]JNJ‐TDP43‐1 in rat and rhesus macaque. (A) Axial and coronal PET images averaged over four‐time windows (0–5, 5–10, 10–30, and 30–60 minutes) in WT rat (left). Time‐activity curve (SUV, g/mL) from the whole brain of [^18^F]JNJ‐TDP43‐1 from 0 to 60 minutes postinjection is shown on the right. (B) Orthogonal SUV images (0–30 minutes, middle; and 60–180 minutes, bottom row) are shown in cross‐sectional view following intravenous bolus injection of [^18^F]JNJ‐TDP43‐1. The subject's normalized MR image is shown for anatomical reference (top row, left). TACs (SUV, g/mL) from representative brain regions of [^18^F]JNJ‐TDP43‐1 from 0 to 240 minutes postinjection are shown on the right.

The PK profile of [^1^ ^8^F]JNJ‐TDP43‐1 was initially characterized in rats before proceeding to radiotracer PK evaluation in rhesus macaque. To evaluate brain permeability, in vivo stability (including potential defluorination), and the PK of [^18^F]JNJ‐TDP43‐1, a dynamic PET scan was performed in WT Sprague‐Dawley rats. As shown in Figure 6a, PET/CT images were averaged across four‐time windows (0–5, 5–10, 10–30, and 30–60 minutes) to illustrate tracer uptake and washout in the rat brain. [^1^ ^8^F]JNJ‐TDP43‐1 demonstrated robust and fast brain penetration, reaching a peak SUV of 1.5 g/mL (*n* =  2) within 2 minutes, followed by rapid and nearly complete washout (> 70%) within 60 minutes, indicating minimal NSB and no detectable defluorination. Then [^1^ ^8^F]JNJ‐TDP43–1 was further evaluated in rhesus macaque to assess the brain permeability and PK profile. The corresponding PET images and TACs are shown in Figure 6b. An initial evaluation of [^18^F]JNJ‐TDP43‐1 was conducted with imaging over a 240 min period. The tracer readily entered the brain, reaching a peak whole brain SUV of 1.7 g/mL (∼3.1% injected dose) at 10 min and exhibiting heterogeneous distribution with reversible kinetics. As shown by the TACs in Figure 6b, no off‐target binding was detected in any brain region examined, and the tracer demonstrated continuous washout over the 240 min imaging period. Tracer uptake was highest in the medial dorsal nuclei, cerebellar white matter, cingulate cortices, and cerebellar cortex, whereas the lowest uptake was observed in the pons and cortical white matter (Figure S3). In the early time frames, there appears to be modest tracer signal in cingulate gyrus, the area commonly affected by FTD. However, like the rest of the cortex, the cingulate region washes out substantially by the later frames. There is no obvious preferential retention or “hot spot” in the cingulate relative to surrounding cortical regions, which suggests the tracer, does not show selective binding or trapping in the cingulate cortex of the normal NHP. Additionally, [^18^F]JNJ‐TDP43‐1 was metabolized rapidly, with 20% and 15% of the parent compound remaining at 30 minutes and 180 minutes, respectively (Figure S4), demonstrating steady activity levels in total plasma and blood beyond 60 minutes. The two‐tissue compartment model (2TCM) fit the data well and yielded a V_T_ of 27.5  ±  2.8 mL/cm^3^ (see Table S3).

### 3.7 In vivo PET assessment of target binding in a disease model

**FIGURE 7:**
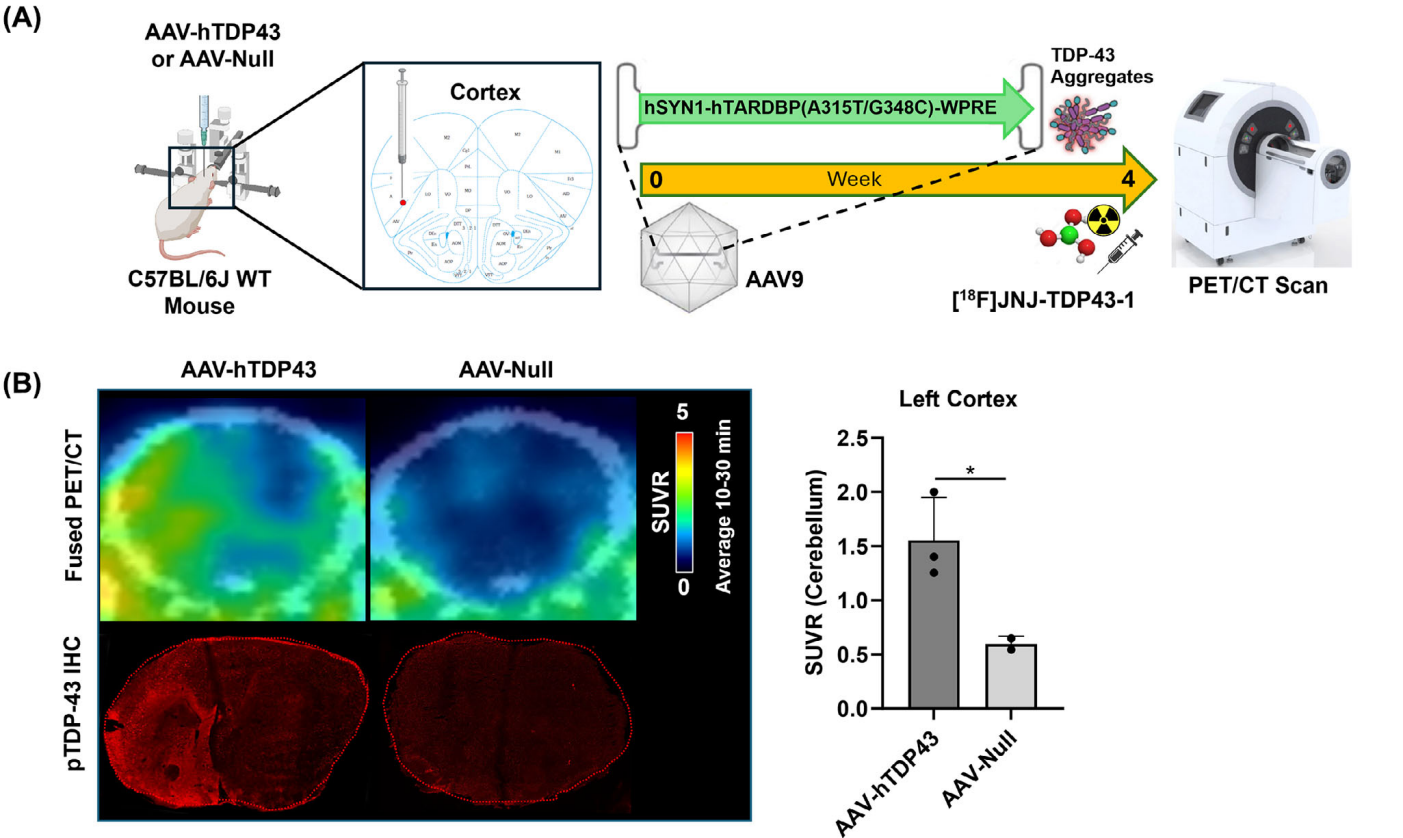
In vivo PET evaluation of tracer binding in a TDP‐43 aggregation mouse model. (A) Schematic overview of the in vivo model used to evaluate tracer binding to TDP‐43 aggregates. C57BL/6J wild‐type mice received cortical injections of either AAV‐hTDP43 or AAV‐Null (control). Over a four‐week period, AAV9‐driven human TDP‐43 expression generated pathological aggregates, and mice were then imaged with [^18^F]JNJ‐TDP43‐1 to evaluate tracer uptake in these regions. (B) Representative PET images (10‐ to 30‐minute average) show elevated tracer uptake in the AAV‐hTDP43‐injected hemisphere compared with the AAV‐Null control. Corresponding pTDP‐43 IHC reveals robust TDP‐43 pathology in AAV‐hTDP43‐injected cortex, with minimal staining in AAV‐Null controls (left). Cerebellum‐normalized SUVR measurements comparing tracer uptake in the AAV‐hTDP43 left hemisphere versus AAV‐Null are shown on the right (Welch's t‐test, *0 < 0.05).

To evaluate the in vivo binding of [^18^F]JNJ‐TDP43‐1, a mouse model expressing pathological human TDP‐43 was generated through intracranial injection of AAV‐hTDP43 or AAV‐Null into the left frontal cortex (Figure 7a). PET imaging was performed at four weeks postinjection. As shown in Figure 7b, in vivo PET imaging with [^18^F]JNJ‐TDP43‐1 revealed markedly elevated tracer uptake in the AAV‐hTDP43‐injected hemisphere compared with the contralateral side. PET images (10‐ to 30‐minute average) showed a clear asymmetry, with higher uptake corresponding to the region expressing pathological TDP‐43. This pattern aligned with pTDP‐43 IHC, which demonstrated robust pTDP‐43 accumulations in the AAV‐hTDP43‐treated cortex and minimal staining in AAV‐Null mice. Quantification of cerebellum‐normalized SUVR confirmed significantly greater tracer uptake in the AAV‐hTDP43 left hemisphere relative to AAV‐Null (SUVR of 1.5 ± 0.4 vs. 0.6 ± 0.07 in the left hemisphere) (Figure 7b).

### 3.8 Off‐target binding and stability measurements

A kinase panel screening of 378 targets was performed using two concentrations of JNJ‐TDP43‐1 (1 µM and 10 µM). See Materials S5.1. No off‐target binding was observed (Table S4). A 75‐target CNS CEREP off‐target panel screen was also conducted, and two off‐targets, human histamine H2 and serotonin 5‐HT5A receptors, showed a blocking effect > 70% at 1 µM (Table S5). To confirm these results, an SPR assay was performed with anti‐H2 or 5‐HT5A polyclonal antibodies in normal human brain homogenates (Materials S5.2). JNJ‐TDP43‐1 showed no interaction with either receptor (Figure S5). Positive controls measured using the same HC brain sample confirmed assay performance: H2 with cimetidine (K_d_ = 159 nM) and 5‐HT5A with serotonin (K_d_ = 1.7 nM).

In vivo radiometabolite measurement of [^18^F]JNJ‐TDP43‐1 in two male Sprague‐Dawley rats revealed no detectable radioactive metabolites in brain tissue 30 minutes after tracer injection (Materials S6 and Figure S6). HPLC analysis of the plasma supernatant demonstrated that 74 ± 5% of the circulating radioactivity corresponded to the parent compound, with the remaining metabolites being significantly more hydrophilic than the parent tracer. JNJ‐TDP43‐1 showed moderate metabolic stability in human (t_1/2_: 168 minutes) and monkey hepatocytes (t_1/2_: 80.5 minutes), but low stability in rat hepatocytes (t_1/2_: 25.0 minutes). See Materials S7 and Table S6 for details.

## 4 DISCUSSION

The development of a PET tracer targeting TDP‐43 inclusions represents a significant breakthrough in the study and diagnosis of NDs associated with TDP‐43 pathology, including ALS, FTD, LATE, and AD. Our findings demonstrate the promising properties of [^18^F]JNJ‐TDP43‐1 as a selective, high‐affinity ligand for TDP‐43 aggregates, with implications for improved diagnostic and therapeutic strategies.

Direct binding assays were utilized to evaluate the compound's affinity, due to the lack of validated reference ligands for pathological TDP‑43. On human brain sections, the autofluorescent JNJ‐TDP43‐1 signal colocalized with pathological TDP‐43 identified by pTDP‐43 IHC and showed no overlap with Aβ, tau, or *α*‐syn, supporting selective target engagement at the cellular level. FTLD‐TDP subtype‐specific measurements are of significant interest; however, they were not incorporated into this study. Future research may employ fluorescent compounds to systematically validate subtype binding, thereby elucidating the diagnostic utility of this tracer in TDP‐43–related pathologies.

Rather than a filter‐binding assay commonly used to estimate K_d_, SPR assay offers label‐free, real‐time monitoring both association and dissociation phases without radiolabels or fluorescent tags. Using the highly sensitive Biacore S200 system, which is capable of detecting small molecule interactions, SPR directly quantified JNJ‐TDP43‐1 binding to pathological TDP‐43. The compound showed consistent high‐affinity binding across 15 brain samples, with an average K_d_ below 10 nM. ARG determined an IC_50_ of 10 nM, confirming the tracer's potency. Binding affinity did not significantly vary across disease samples, including FTLD‐TDP, ALS, ALS/FTD, and LATE‐NC/ADNC (K_d_ ranged from 3.9 to 8.4 nM). ARG competition assays further confirmed strong, displaceable binding on ALS, FTLD‐TDP, and LATE‐NC/ADNC sections. Together, these results indicate that the tracer recognizes a shared pathological TDP‐43 conformer across different TDP‐43 related NDs.

Despite known differences in regional distribution, morphology, and molecular processing of TDP‐43 aggregates among ALS, FTLD‐TDP and LATE‐NC/ADNC, the consistent binding profile highlights the robustness of JNJ‐TDP43‐1 and supports its potential as an applicable in vivo biomarker for pathological TDP‐43. The compound also displays excellent selectivity over Aβ, tau, and *α*‐syn aggregates, with K_d_ values all exceeding 8000 nM. This affinity is not only indicative of the tracer's potential efficacy in detecting TDP‐43 pathology but also confirms its selectivity over other aggregated proteins, which is crucial for minimizing NSB and enhancing signal clarity in PET imaging.

When ARG and pTDP‐43 IHC were compared side‐by‐side on adjacent sections, the colocalization of ARG signals with pTDP‐43 immunostaining across multiple brain regions confirmed the tracer's ability to detect pathological TDP‐43 in human FTLD‐TDP brain tissue. Furthermore, the tracer appears to bind to FTLD‐TDP subtypes A and B (refer to Figure 4a). Nevertheless, comprehensive analysis of ARG binding based on immunohistochemically defined subtypes is required to substantiate evidence for FTLD‐TDP subtype‐specific pathology. The same assay conducted on mouse brain sections injected with AAV‐hTDP43 revealed a similar pattern of ARG distribution when compared to the pathological distribution of TDP‐43. These findings substantiate the tracer's in vitro target engagement.

The radiosynthesis of [^18^F]JNJ‐TDP43‐1 is straightforward via a one‐step nucleophilic aromatic substitution reaction. The tracer could be produced consistently with good radiochemical yields, excellent radiochemical purity, and high molar activity. In vivo PET studies demonstrated a favorable PK profile for [^18^F]JNJ‐TDP43‐1, supporting its suitability for translation into human studies. Across WT rats and nonhuman primates, the tracer showed robust brain uptake and rapid washout kinetics, features that may enhance the signal‐to‐noise ratio during imaging. Importantly, in the TDP‐43 aggregate mouse model, elevated tracer uptake in the AAV‐hTDP43‐injected hemisphere closely colocalized with pTDP‐43 pathology, providing the first clear demonstration of in vivo PET tracer binding to TDP‐43 aggregates. Together, these findings highlight [^18^F]JNJ‐TDP43‐1 as a promising candidate for further development toward clinical imaging of TDP‐43 proteinopathies.

In cell‐based off‑target screening, JNJ‑TDP43‑1 demonstrated a blocking effect greater than 70% at 1 µM against the 5‐HT5A and H2 receptors. However, follow‐up SPR analysis directly assessed JNJ‐TDP43‐1 binding to both receptors and found no evidence of specific interaction, as the sensorgrams fit poorly and did not yield reliable $K_{d}$ values (Figure S5). Moreover, the tracer was stable in both in vivo radiometabolite and in vitro hepatocyte studies. Overall, JNJ‐TDP43‐1 showed minimal off‐target binding, with no major metabolites detected.

A recently published first‐in‐class TDP‐43 PET tracer from AC Immune, [^1^ ^8^F]ACI‐19626, provides an important benchmark for the field. Although [^1^ ^8^F]ACI‐19626 demonstrated binding affinity toward aggregated TDP‐43 with reported K_d_ values of 20–25 nM in FTLD‐TDP tissue, the tracer failed to show displaceable autoradiographic signals in ALS brain sections, indicating limited sensitivity to certain TDP‐43 aggregate types. In contrast, [^1^ ^8^F]JNJ‐TDP43‐1 exhibited consistently higher affinity (K_d_ = 7.1 nM) across ALS, FTLD‐TDP, and LATE‐NC/ADNC samples, along with strong, displaceable ARG signals even in brain regions with relatively low pathological burden. Moreover, while in vivo evaluation of [^1^ ^8^F]ACI‐19626 in a disease‐relevant animal model has not yet been reported, our tracer demonstrated robust and selective in vivo binding in an AAV‐driven TDP‐43 mouse model. Elevated tracer retention clearly colocalized with pTDP‐43 expression, providing direct evidence of in vivo target binding. Although the A315T and G348C mutations are primarily associated with ALS/FTLD, this model recapitulates key features of TDP‐43 dysfunction relevant across neurodegenerative diseases. The AAV‐driven approach enables controlled, region‐specific induction of pathology, facilitating the study of protein mislocalization, aggregation, and synaptic dysfunction in vivo, and providing a mechanistically informative and translationally relevant platform for evaluating TDP‐43 targeted imaging. Together, these results highlight the superior sensitivity and validated in vivo performance of [^1^ ^8^F]JNJ‐TDP43‐1 relative to the AC Immune tracer, underscoring its strong potential as a next‐generation imaging agent for TDP‐43 proteinopathies. Table S7 contains a side‐by‐side comparison of the tracers [^1^ ^8^F]JNJ‑TDP43‑1 and [^1^ ^8^F]ACI‑19626.

## 5 CONCLUSIONS

The significance of imaging TDP‐43 pathology cannot be overstated, as it is increasingly acknowledged that TDP‐43 proteinopathies are present not only in ALS, FTLD, LATE but also play a role in the cognitive dysfunction associated with AD9.^25^ Current diagnostic methods primarily rely on clinical assessments and *post mortem* evaluations, which can result in delays in therapy initiation. By facilitating early diagnosis through non‐invasive imaging, [18F]JNJ‐TDP43‐1 could change the landscape of disease management and monitoring. In addition to aiding in early diagnosis, this tracer opens avenues for monitoring disease progression and evaluating therapeutic efficacy. For patients with TDP‐43 related disorders, the ability to visualize changes in TDP‐43 aggregation in real time can provide insights into disease dynamics, informing treatment decisions, and potentially improving patient outcomes.

In conclusion, the development of [^18^F]JNJ‐TDP43‐1 marks a significant advancement in imaging TDP‐43‐related NDs. Its validated binding affinity and specificity offer an exciting opportunity to improve our understanding of TDP‐43 pathology, ultimately facilitating better diagnostic and therapeutic strategies for combating these challenging conditions. Future work will further explore its clinical applicability and use in real‐world settings, with the hope that it can contribute to improving the lives of those affected by TDP‐43 related diseases.

## AUTHOR CONTRIBUTIONS

**Chunfang A. Xia**: Conceptualization; formal analysis; investigation; methodology; project administration; visualization; writing—original draft; writing—review and editing. **Mani Salarian**: Formal analysis; investigation; writing—original draft; writing—review and editing. **Christopher J. Gartshore**: Formal analysis; investigation. **Antonella Scaglione; Thomas Hayes; Shuanglong Liu; Hsiu‐Ming Tsai; Javier Echavarren; Alessandra Matzeu**: Investigation. **Jose Maria Cid**: Conceptualization; methodology. **A. Katrin Szardenings**: Conceptualization; writing—review and editing.

## CONFLICT OF INTEREST STATEMENT

The authors declare no conflicts of interest. Author disclosures are available in the Supporting Information.

## CONSENT STATEMENT

Not applicable.

## Supporting information



Supporting Information

Supporting Information

## Data Availability

The data supporting the findings of this study are available within the article and/or its supplementary information.
