# Supplementary material for: Development and characterization of a novel TDP‐43 positron emission tomography tracer: [18F]JNJ‐TDP43‐1
Source: Alzheimers Dement. 2026 Jul 18;22(7):e71675. doi: 10.1002/alz.71675 (PMC13380671; doi:10.1002/alz.71675)
Supplement: Supplementary file 1 — Supporting Information [file ALZ-22-e71675-s001.docx]

**Supplementary**

**Title: Development and Characterization of a Novel TDP-43 Positron Emission Tomography Tracer: [^18^F]JNJ-TDP43-1**

**Supplementary Materials**

1. **Subcellular fractionation preparation of post-mortem human brain samples for western blot (WB) analysis and surface plasmon resonance (SPR) assay**

Post-mortem human brain samples were prepared for WB and SPR assay. For subcellular protein fractionation, tissue homogenates were processed using the Subcellular Protein Fractionation Kit for Tissues (ThermoFisher Scientific, MA, Cat# 87790) according to the manufacturer’s protocol with minor modifications. Briefly, frozen brain tissue was mechanically disrupted and homogenized in ice-cold extraction buffer supplemented with protease inhibitors. Homogenates were cleared by filtration and low-speed centrifugation to remove insoluble debris and nuclei. An aliquot of the initial homogenate was retained as the whole fraction (WF). The remaining material was sequentially extracted to enrich subcellular fractions, including the cytoplasmic fraction obtained with cytoplasmic extraction buffer, the nuclear fraction obtained with nuclear extraction buffer, the cell membrane fraction (CM) obtained with membrane extraction buffer, and the chromatin-binding portion (CBP) recovered from the nuclear pellet after chromatin extraction. All fractions were collected on ice, clarified by centrifugation as recommended, and total protein concentrations were determined using the Pierce™ BCA Protein Assay Kit (ThermoFisher Scientific Inc., MA) prior to downstream WB and SPR analyses.

1. **Synthesis of JNJ‑TDP43‑1, as shown in Supplementary Figure 1**

All materials were purchased from commercial suppliers and used without further purification. [^18^F]fluoride was obtained from PETNET (Loma Linda, CA). Radiochemical synthesis was performed using a Synthra RNPlus radiosynthesis module (Synthra, Hamburg, Germany), and analytical HPLC was conducted on an Agilent HPLC system equipped with a radiodetector.

- 1. **2-chloro-4-(3-ethylazetidin-1-yl)quinazoline, 3.**

A solution of DIPEA (3.588 mL, 20.599 mmol) in acetonitrile (20 mL) was added dropwise over 5 min to a cold (0 °C) suspension of 2,4‑dichloroquinazoline (0.410 g, 2.060 mmol) and 3‑ethylazetidine trifluoroacetate (0.451 g, 2.266 mmol) in acetonitrile (20 mL) under nitrogen. The resulting mixture was stirred at 0 °C for 2 hr, then diluted with ethyl acetate and washed with water. The organic phase was dried over anhydrous MgSO4, filtered and concentrated in vacuo to afford **3** as a beige solid (0.424 g, 79%), which was used in the subsequent step without further purification. Mass spectrum (ESI, m/z): Calcd. for C13H14ClN3, 247.1, found [M+H]+, 248.2. 1H NMR (300 MHz, DMSO-d_6_) d 7.98 (d, J = 8.3 Hz, 1H), 7.78 (ddd, J = 8.3, 7.0, 1.3 Hz, 1H), 7.64 – 7.59 (m, 1H), 7.47 (ddd, J = 8.3, 7.0, 1.2 Hz, 1H), 5.07–3.68 (m, 4H), 2.80–2.63 (m, 1H), 1.67 (p, J = 7.3 Hz, 2H), 0.90 (t, J = 7.3 Hz, 3H).

**2.2 4-(3-ethylazetidin-1-yl)-N-((6-fluoropyridin-3-yl)methyl)quinazolin-2-amine, JNJ-TDP43-1.**

A suspension of (6‑fluoropyridin‑3‑yl) methanamine (0.285 g, 2.261 mmol) and **3** (0.140 g, 0.565 mmol) in isopropyl alcohol (7 mL) was placed in a microwave vial, sealed, and heated at 150 °C for 1 h under microwave irradiation. The resulting orange mixture was diluted with ethyl acetate (30 mL) and washed with water (30 mL). The organic layer was dried over anhydrous MgSO_4_, filtered and concentrated in vacuo to afford an orange solid. Purification by silica gel chromatography (12 g, irregular 40–60 μm silica; gradient 0–10% MeOH/DCM) yielded JNJ‑TDP43‑1 as a beige solid (0.087 g, 45%). Calcd. for C_19_H_20_FN_5_, 337.1703, found [M+H]^+^, 338.1562. 1H NMR (400 MHz, DMSO-d_6_) d ppm 0.88 (t, J=7.30 Hz, 3 H) 1.63 (quin, J=7.41 Hz, 2 H) 2.60 - 2.69 (m, 1 H) 4.01 (br s, 2 H) 4.49 (br d, J=6.20 Hz, 4 H) 6.98 - 7.03 (m, 1 H) 7.10 (dd, J=8.44, 2.72 Hz, 1 H) 7.16 - 7.23 (m, 1 H) 7.26 (br d, J=8.30 Hz, 1 H) 7.44 - 7.52 (m, 1 H) 7.71 (d, J=8.11 Hz, 1 H) 7.95 (td, J=8.30, 2.48 Hz, 1 H) 8.21 (s, 1 H).

**2.3 N-((6-chloropyridin-3-yl)methyl)-4-(3-ethylazetidin-1-yl)quinazolin-2-amine, JNJ-TDP43-2.**

(6-chloropyridin-3-yl) methanamine (0.280 g, 1.130 mmol), and **3** (0.645 g, 4.521 mmol) were suspended in isopropyl alcohol (15 mL) in a microwave vial. The vial was sealed and stirred at 150 °C for 1 h under microwave irradiation. The orange reaction mixture was diluted with ethyl acetate (75 mL) and washed with water (50 mL). The organic layer was dried over anhydrous MgSO_4_, filtered and concentrated to dryness in vacuo to give an orange solid. The solid was then subjected to silica gel chromatography (25 g, irregular 40-60 µm silica, gradient 0-10% MeOH/dichloromethane, yielding JNJ-TDP43-2 (0.286 g, 71 %) as a white solid. Calcd. for C19H20ClN5, 353.1407, found [M+H]+, 354.1262. 1H NMR (400 MHz, DMSO-d6) 8.40 (d, 1H, J=2.2 Hz), 7.82 (dd, 1H, J=2.4, 8.2 Hz), 7.72 (d, 1H, J=8.3 Hz), 7.49 (t, 1H, J=7.2 Hz), 7.44 (d, 1H, J=8.2 Hz), 7.26 (br d, 2H, J=8.1 Hz), 7.02 (t, 1H, J=7.6 Hz), 4.49 (br d, 4H, J=6.2 Hz), 4.01 (br s, 2H), 2.5-2.7 (m, 1H), 1.64 (quin, 2H, J=7.4 Hz), 0.89 (t, 3H, J=7.3 Hz).

1. **Radiosynthesis of JNJ-TDP43-1, as shown in Supplementary Figure 1**

**4-(3-ethylazetidin-1-yl)-N-((6-(fluoro-[^18^F])pyridin-3-yl)methyl)quinazolin-2-amine,[^18^F]JNJ-TDP43-1.**

[^18^F]fluoride was trapped on a QMA ion-exchange cartridge, and a solution of potassium carbonate (3 mg) and Kryptofix 222^®^ (15 mg) in 9:1 acetonitrile/water was used to elute the fluoride into a reactor. The eluate was azeotropically dried at 80 °C under vacuum with a stream of nitrogen. Additional acetonitrile (1 mL) was then added, and the mixture was further evaporated at 100 °C for 5 min and subsequently at 110 °C for 3 min. After cooling to 50 °C, a solution of JNJ‑TDP43‑2 (2.9–4.2 mg) in anhydrous DMSO (0.75 mL) was introduced into the reactor, and the reaction mixture was heated at 180 °C for 10 min. The reaction was cooled to 50 °C, diluted with 25% acetonitrile in water (3.5 mL) and purified by semi-preparatory HPLC (Agilent Eclipse XDB C18, 250 × 9.4 mm; 45% acetonitrile in 10 mM NH₄OAc, 5 mL/min). Fractions containing [^18^F]1 were collected into 35 mL of water and then isolated on a C18 Sep-Pak light cartridge. Purified [¹⁸F]JNJ‑TDP43‑1 was eluted with 0.5 mL ethanol, formulated with 4.5 mL saline, and transferred to the final product vial. Identity and purity were confirmed by analytical HPLC (Kinetex EVO 5 µm, C18, 150 × 4.6 mm; mobile phase A = acetonitrile + 0.1% TFA; mobile phase B = water + 0.1% TFA; gradient: 0–0.5 min 5% A, 0.5–9 min 5–50% A, 9–9.1 min 50–90% A, 9.1–12 min 90% A). The radiochemical yield was 28% ± 7% (n = 9, decay‑corrected to start of synthesis), and the molar activity was 52 ± 14 GBq/µmol.

1. **WB analysis of TDP-43 pathology in brain homogenates**

Simple Western kits for Jess (ProteinSimple, San Jose, CA) were purchased. pTDP-43 protein levels in different subcellular fractionations were measured following the manufacturer’s instructions. The standard pack reagents were prepared. Tissue samples were mixed with 0.1x Sample Buffer to a final concentration of 1 µg/µL, reduced and denatured. The prepared samples, primary (see Supplementary Table 2) and secondary antibodies (ProteinSimple, San Jose, CA) and chemiluminescent substrate were dispensed in microliter volumes into designated wells in an assay plate from a 12-230 kDa Wes separation module kit. Then the capillary cartridge and the prepared assay plate were placed in the Jess for an automated analysis. The molecular weight and signals for immunodetected proteins were automatically reported. Total protein was also measured at the same time for sample loading normalization. Compass for SW (ProteinSimple, San Jose, CA) was used to report molecular weight, area, and percent area for each protein detected.

Western blot analysis showed that pTDP-43 is mainly found in the cytoplasm compared to other subcellular fractions (Supplementary Figure 2a). Then the cytoplasmic samples from human brain specimens were used to evaluate pathological TDP-43 inclusions. Disease samples displayed higher pTDP-43 levels than normal controls, as depicted in Supplementary Figure 2b.

1. **Off-target binding assays**

**5.1 Off-target screening:** This cell‑based in vitro pharmacology screen assesses potential interactions of small molecules with relevant kinases, receptors, transporters, ion channels, and enzymes. JNJ-TDP43-1 was screened against 378 kinases (1 µM and 10 µM) and no off-target binding detected (see Supplementary Table 4). Off-target activity across 75 CNS targets was also measured and the result is shown in Supplementary Table 5. Both studies were performed by Eurofins Discovery, Celle‑Lévescault, France.

**5.2  SPR K_d_ measurement for 2 off-targets (H2 and 5-HT5A) in healthy control (HC) human brain homogenates:** JNJ‑TDP43‑1 demonstrated > 70% blocking effect to 5‑HT5A and H₂ receptors at 1 µM in the CEREP panel screening assay (see Supplementary Table 5). Binding of JNJ‑TDP43‑1 to human histamine H2 and serotonin 5‑HT5A receptors in HC brain homogenates was assessed by SPR, as described in Methods 2.2 of the main manuscript. The H2 and 5‑HT5A receptors were captured via their respective polyclonal antibodies (See Supplementary Table 2). SPR sensorgrams showed poor curve fitting and no significant binding of JNJ‑TDP43‑1 to either receptor, as summarized in Supplementary Figure 5. Positive controls in the same HC brain sample confirmed assay performance: H2 with cimetidine (K_d_ = 159 nM) and 5-HT5A with serotonin (K_d_ = 1.7 nM).

1. **In vivo radiometabolite measurement of [^18^F]JNJ-TDP43-1**

Two male Sprague–Dawley rats (280-300 g) were anesthetized with 1–2% isoflurane in oxygen, followed by intravenous bolus of ~25.9 MBq of [^18^F]JNJ-TDP43-1 in 300 μL saline containing < 5% ethanol. At 30 min post-injection, brain and whole blood samples were collected. Approximately 500 µL of whole blood was drawn into microtainer tubes containing K₂EDTA (BD Biosciences, USA) and divided into three 150 µL aliquots. One aliquot was retained for gamma counting, while the remaining two were centrifuged for 5 min at 13,000 × g. The plasma supernatant was transferred to fresh centrifuge tubes, and cold acetonitrile (100 µL) was added to precipitate plasma proteins. After trituration, samples were centrifuged again for 5 min at 13,000 × g, and the supernatant was collected. Red blood cell, plasma protein, and plasma supernatant fractions were counted by a gamma counter (Wizard 1470, PerkinElmer, MA). A second plasma‑supernatant sample was diluted with water (350 µL), and 480 µL was analyzed by gradient reverse‑phase HPLC (mobile phase A: 0.1% formic acid in water; mobile phase B: acetonitrile; gradient: 0–1 min, 5% B; 1–10 min, 5–95% B; 10–12 min, 95% B; then returned to 5% B).

Brains were collected and homogenized in a glass tissue grinder with 2 mL of acetonitrile/water. Three 300 µL aliquots were transferred to centrifuge vials; one was retained for gamma counting, and the other two were centrifuged for 5 min at 13,000 × g. Supernatants were separated, and one was diluted with 350 µL water and spiked with 20 µL of cold JNJ‑TDP43‑1 solution. Whole‑brain homogenate, separated tissue, and supernatant fractions were quantified by gamma counting, and the area under the HPLC radio‑chromatogram curve was used to determine the extent of [¹⁸F]JNJ‑TDP43‑1 metabolism.

Separation of the brain homogenates into tissue and supernatant fractions followed by HPLC analysis revealed no detectable radioactive metabolites in the brain tissue (Supplementary Figure 6, middle). Between 35 and 49% of the total activity in the brain homogenate was recovered in the supernatant, indicating a substantial proportion of tracer unbound to brain tissue. Gamma counting further showed that most radioactive species were not associated with red blood cells or plasma proteins, with 64% of total activity residing in the plasma‑supernatant fractions. Red blood cell fractions accounted for 24–27% of total activity, while 7–11% of the radioactivity was associated with plasma proteins. HPLC analysis of the plasma supernatant demonstrated that 73 ± 5% of the circulating radioactivity corresponded to the parent compound [¹⁸F]JNJ‑TDP43‑1, with the remaining metabolites being significantly more hydrophilic than the parent tracer.

1. **Determination of JNJ-TDP43-1 stability in human, monkey and rat hepatocytes**

The metabolic stability of JNJ-TDP43-1 was evaluated in cryopreserved human, cynomolgus monkey, and rat hepatocytes. Hepatocytes were thawed, assessed for viability, and suspended in prewarmed incubation medium at a final density of approximately 0.5 × 10^6^ viable cells/mL. JNJ-TDP43-1 was added to the hepatocyte suspensions at a final concentration of 1 µM, and incubations were performed at 37 °C with gentle shaking. Aliquots were collected at predetermined time points and quenched with ice-cold acetonitrile containing internal standard. Samples were centrifuged to remove precipitated protein, and supernatants were analyzed by LC-MS/MS to quantify the remaining parent compound. The percentage of parent compound remaining was plotted versus incubation time, and in vitro half-life (t_1/2_) was calculated from the first-order depletion rate constant. Assay performance was confirmed using the reference control compounds midazolam and diclofenac. The results are summarized in Supplementary Table 6.

**Supplementary Tables**

**Supplementary Table 1.** Human brain sample information and co-pathologies measured by IHC.

**Standard IHC Scoring System:**

0 = none (no specific inclusions); 1 = mild (rare/sparse inclusions; focal); 2 = moderate (readily identifiable; multifocal); 3 = severe (frequent/widespread; confluent fields).

Abbreviations: HC, healthy control; FTLD-TDP, frontotemporal lobar degeneration with TDP-43 inclusions; ALS, amyotrophic lateral sclerosis; FTD, frontotemporal dementia; SPR, surface plasmon resonance; ARG, autoradiography; IHC, immunohistochemistry; FCS, fluorescent compound staining; ADNC, Alzheimer’s disease neuropathologic change; LATE-NC, limbic-predominant age-related TDP-43 encephalopathy neuropathologic change; TDP-43, TAR DNA-binding protein 43; Aβ, beta-amyloid; α-syn, alpha-synuclein.

**Supplementary Table 2: Antibodies used in immunohistochemistry**

| **Target** | **Antibody** | **Assay** | **Vendor** | **Cat #** | **Clone** |
| --- | --- | --- | --- | --- | --- |
| TDP-43 | pTDP-43 | IHC/FCS | Invitrogen | #114661 | Ser409/410 |
|  | pTDP-43 | WB/SPR | ProteinTech | #22309-1-AP | Ser409/410 |
| Aβ | Aβ42 | SPR | CreativeBiolabs | PABL-011 | 3D6 |
|  | Aβ42 | IHC | abcam | ab201060 | mOC64 |
| tau | p-tau | SPR | JNJ | JNJ-3657 | pT3 |
| a-syn | p-syn | SPR | abcam | ab51253 | pS129 |
|  | p-syn | IHC | Invitrogen | #180215 | pS129 |
| H2 | H2 | SPR | ThermoFisher | HA500154 | polyclonal |
| 5-HT5A | 5-HT5A | SPR | ThermoFisher | BS-12055R | polyclonal |

Abbreviations: pTDP-43, phosphorated TAR DNA-binding protein 43; Aβ, Beta-amyloid; p-tau, phospho-tau; α-syn, alpha-synuclein; IHC, immunohistochemistry; SPR, surface plasmon resonance; WB, western blot; JNJ, Johnson & Johnson; H2, histamine H2 receptor; 5-HT5A, serotonin 5-hydroxytryptamine receptor 5A.

**Supplementary Table 3.** Regional distribution volume (V_T_, mL/cm³) of [¹⁸F]JNJ‑TDP43‑1 in non‑human primates (NHP).

| **Region** | **V_T_ (mL/cm^-3^)** |
| --- | --- |
| Frontal lobe | 26.6 |
| Temporal lobe | 26.5 |
| Hippocampus | 27.1 |
| Amygdala | 26.8 |
| Parietal lobe | 25.8 |
| Occipital lobe | 24.2 |
| Anterior Cingulate | 31.2 |
| Isthmus Cingulate | 26.0 |
| Posterior Cingulate | 31.8 |
| Insula | 29.3 |
| Claustrum | 28.9 |
| Caudate Nucleus | 24.8 |
| Putamen | 28.9 |
| Ventral Striatum | 26.2 |
| Basal forebrain | 26.8 |
| Globus Pallidus | 27.9 |
| Thalamus | 28.8 |
| Hypothalamus | 23.4 |
| Cerebellar lobes | 28.7 |
| Cerebellar nuclei | 30.6 |
| Vermis | 32.3 |
| Cortical WM | 27.0 |
| Cerebellar WM | 33.2 |
| Midbrain | 25.9 |
| Pons | 21.9 |
| Medulla | 26.5 |
| Brainstem | 24.3 |
| Anterior Cingulate, hot | 29.8 |
| Straight Gyrus | 29.1 |
| Presubiculum | 27.0 |
| Medial Dorsal Nucleus | 34.2 |

**Supplementary Table 4.** A kinase panel screening of 378 targets was performed using two concentrations of JNJ-TDP43-1 (1 µM and 10 µM). No off-target binding was observed.

| **Kinase** | **% of Inhibiton** | | **Kinase** | **% of Inhibiton** | |
| --- | --- | --- | --- | --- | --- |
| AAK1(h) | 1 µM | 10 µM | MEKK2(h) | -19 | -9 |
| Abl(h) | -1 | -3 | MEKK3(h) | -2 | -1 |
| ACK1(h) | 6 | -1 | MELK(h) | -1 | 3 |
| ACTR2(h) | -6 | 11 | Mer(h) | 7 | 4 |
| ALK(h) | -3 | 0 | Met(h) | 4 | -1 |
| ALK1(h) | 6 | 7 | MINK(h) | 3 | -28 |
| ALK2(h) | -22 | -22 | MKK3(h) | -2 | -5 |
| ALK4(h) | -7 | -3 | MKK6(h) | 7 | 7 |
| ALK6(h) | -9 | -5 | MLCK(h) | -7 | 4 |
| Arg(h) | -6 | 3 | MLK1(h) | 3 | 1 |
| AMPKα1(h) | -1 | -23 | MLK2(h) | -1 | 3 |
| AMPKα2(h) | 4 | 0 | MLK3(h) | -4 | -2 |
| A-Raf(h) | -8 | -21 | MLK4(h) | 3 | 0 |
| ARK5(h) | -1 | -3 | Mnk1(h) | 0 | 7 |
| ASK1(h) | -2 | -10 | Mnk2(h) | 19 | -23 |
| Aurora-A(h) | -1 | -4 | MOK(h) | -6 | -11 |
| Aurora-B(h) | 11 | 6 | MRCKα(h) | -9 | -14 |
| Aurora-C(h) | -14 | -9 | MRCKβ(h) | -3 | -11 |
| Axl(h) | 2 | -4 | MRCKγ(h) | -7 | -2 |
| BIKe(h) | 5 | 7 | MSK1(h) | -4 | -12 |
| Blk(h) | 11 | 14 | MSK2(h) | -7 | -8 |
| BMPR2(h) | 10 | 2 | MSSK1(h) | -6 | -19 |
| Bmx(h) | -9 | -7 | MST1(h) | -8 | -28 |
| BRK(h) | 12 | 0 | MST2(h) | 6 | 3 |
| BrSK1(h) | -8 | -6 | MST3(h) | -3 | -9 |
| BrSK2(h) | -7 | -12 | MST4(h) | -7 | -8 |
| BTK(h) | -3 | 1 | mTOR(h) | -7 | -19 |
| B-Raf(h) | -3 | -14 | mTOR/FKBP12(h) | 2 | 16 |
| CaMKI(h) | -6 | -11 | MuSK(h) | 8 | 8 |
| CaMKIβ(h) | 2 | 6 | MYLK2(h) | -3 | -13 |
| CaMKIγ(h) | -3 | -8 | MYO3B(h) | -16 | -3 |
| CaMKIIα(h) | 1 | -4 | NDR1(h) | -3 | -2 |
| CaMKIIβ(h) | -2 | -10 | NDR2(h) | -5 | -20 |
| CaMKIIγ(h) | -1 | 8 | NEK1(h) | -11 | -44 |
| CaMKIδ(h) | 2 | 9 | NEK2(h) | -11 | -7 |
| CaMKIIδ(h) | -2 | -7 | NEK3(h) | -6 | 0 |
| CaMKIV(h) | -11 | -5 | NEK4(h) | -11 | -4 |
| CaMKK1(h) | -16 | 16 | NEK5(h) | 0 | -5 |
| CaMKK2(h) | -2 | 11 | NEK6(h) | -1 | -12 |
| Cdc7/cyclinB1(h) | -13 | -8 | NEK7(h) | 6 | -7 |
| CDK1/cyclinB(h) | -4 | -1 | NEK9(h) | -8 | -5 |
| CDK2/cyclinA(h) | 1 | -11 | NIM1(h) | -4 | 6 |
| CDK2/cyclinE(h) | 7 | -8 | NEK11(h) | -10 | -21 |
| CDK3/cyclinE(h) | -2 | -12 | NLK(h) | 2 | -2 |
| CDK4/cyclinD3(h) | -4 | -13 | NUAK2(h) | -7 | -3 |
| CDK5/p25(h) | 0 | -5 | OSR1(h) | -3 | -2 |
| CDK5/p35(h) | 2 | 3 | p70S6K(h) | -1 | -3 |
| CDK6/cyclinD3(h) | -10 | -12 | p70S6Kβ(h) | 7 | 4 |
| CDK7/cyclinH/MAT1(h) | 1 | 1 | PAK1(h) | -3 | -30 |
| CDK9/cyclin T1(h) | 5 | -3 | PAK2(h) | -4 | 2 |
| CDK14/cyclinY(h) | -2 | 2 | PAK4(h) | 4 | 6 |
| CDK16/cyclinY(h) | -12 | -7 | PAK3(h) | 4 | 5 |
| CDK17/cyclinY(h) | -6 | -37 | PAK5(h) | -8 | -11 |
| CDK18/cyclinY(h) | -5 | -2 | PAK6(h) | -5 | -14 |
| CDKL1(h) | -2 | -3 | PAR-1Bα(h) | -11 | -11 |
| CDKL2(h) | 3 | -4 | PASK(h) | -8 | -10 |
| CDKL3(h) | -8 | -12 | PEK(h) | -14 | -21 |
| CDKL4(h) | -9 | 22 | PDGFRα(h) | -4 | -4 |
| ChaK1(h) | -16 | -3 | PDGFRβ(h) | -13 | -12 |
| CHK1(h) | 13 | 1 | PDHK2(h) | 10 | 15 |
| CHK2(h) | -11 | -4 | PDHK4(h) | 6 | -24 |
| CK1α(h) | -2 | -10 | PDK1(h) | -7 | -20 |
| CK1ε(h) | -4 | 2 | PhKγ1(h) | -12 | 15 |
| CK1γ1(h) | -2 | -10 | PhKγ2(h) | -9 | -7 |
| CK1γ2(h) | 0 | -11 | Pim-1(h) | -1 | -6 |
| CK1γ3(h) | 0 | 2 | Pim-2(h) | 2 | 3 |
| CK1δ(h) | -14 | -10 | Pim-3(h) | -9 | -23 |
| CK2(h) | -4 | -8 | PKA(h) | -10 | -9 |
| CK2α1(h) | -3 | -8 | PKAcβ(h) | -1 | 12 |
| CK2α2(h) | 19 | 26 | PKBα(h) | -8 | -8 |
| CLIK1(h) | -25 | -12 | PKBβ(h) | 11 | 4 |
| CLK1(h) | -4 | -10 | PKBγ(h) | -12 | -36 |
| CLK2(h) | -11 | -1 | PKCα(h) | -3 | -8 |
| CLK3(h) | -10 | -2 | PKCβI(h) | 5 | 4 |
| CLK4(h) | 8 | 10 | PKCβII(h) | 0 | 4 |
| cKit(h) | -17 | -18 | PKCγ(h) | -4 | -3 |
| COT(h) | 8 | -14 | PKCδ(h) | 0 | 5 |
| CRIK(h) | -2 | -11 | PKCε(h) | -1 | -3 |
| CSK(h) | 1 | -6 | PKCη(h) | -1 | -15 |
| c-RAF(h) | 1 | -6 | PKCι(h) | -21 | -13 |
| cSRC(h) | -1 | 8 | PKCμ(h) | -14 | -13 |
| DAPK1(h) | -8 | -5 | PKCθ(h) | 3 | -5 |
| DAPK2(h) | -2 | 2 | PKCζ(h) | 2 | -6 |
| DCAMKL1(h) | 5 | -12 | PKD2(h) | -22 | -13 |
| DCAMKL2(h) | -7 | -3 | PKD3(h) | 1 | -2 |
| DCAMKL3(h) | -3 | -41 | PKG1α(h) | 5 | 7 |
| DDR1(h) | -7 | -12 | PKG1β(h) | -12 | -8 |
| DDR2(h) | -6 | -14 | PKR(h) | -2 | 1 |
| DMPK(h) | -4 | -18 | Plk1(h) | -6 | -3 |
| DRAK1(h) | 1 | 8 | Plk3(h) | -6 | -5 |
| DRAK2(h) | 3 | -9 | Plk4(h) | -4 | 0 |
| DYRK1A(h) | -2 | -11 | PRAK(h) | 8 | 16 |
| DYRK1B(h) | 2 | 2 | PRKG2(h) | -2 | 28 |
| DYRK2(h) | -9 | 6 | PRK1(h) | 4 | -16 |
| DYRK3(h) | 2 | -1 | PRK2(h) | 9 | 3 |
| eEF-2K(h) | 3 | 8 | PrKX(h) | -1 | -11 |
| EGFR(h) | -10 | -14 | PRP4(h) | 4 | -15 |
| EphA1(h) | 2 | 3 | PTK5(h) | -11 | -3 |
| EphA2(h) | -3 | 5 | Pyk2(h) | -2 | 3 |
| EphA3(h) | -5 | -2 | Ret(h) | -7 | 4 |
| EphA4(h) | -12 | -35 | RIPK1(h) | -1 | -12 |
| EphA5(h) | -4 | -19 | RIPK2(h) | -3 | 9 |
| EphA7(h) | -6 | -2 | ROCK-I(h) | -4 | -3 |
| EphA8(h) | 3 | -9 | ROCK-II(h) | 2 | -4 |
| EphB2(h) | 3 | -7 | Ron(h) | 3 | 1 |
| EphB1(h) | -14 | -5 | Ros(h) | -11 | -6 |
| EphB3(h) | -1 | -11 | Rse(h) | -6 | -16 |
| EphB4(h) | -23 | -11 | Rsk1(h) | -4 | -14 |
| ErbB2(h) | 2 | 7 | Rsk2(h) | -5 | -34 |
| ErbB4(h) | -7 | -6 | Rsk3(h) | -11 | -19 |
| FAK(h) | -15 | -9 | Rsk4(h) | -3 | -3 |
| Fer(h) | -5 | -8 | SAPK2a(h) | -9 | -4 |
| Fes(h) | -7 | -8 | SAPK2b(h) | 17 | 6 |
| FGFR1(h) | 1 | -10 | SAPK3(h) | -11 | -3 |
| FGFR2(h) | -7 | -6 | SAPK4(h) | 5 | 7 |
| FGFR3(h) | -9 | -8 | SBK1(h) | -9 | -5 |
| FGFR4(h) | 1 | -21 | SGK(h) | -2 | -6 |
| Fgr(h) | -13 | -24 | SGK2(h) | 3 | -16 |
| Flt1(h) | -5 | -12 | SGK3(h) | -2 | -32 |
| Flt3(h) | -9 | -1 | SIK(h) | -1 | -11 |
| Flt4(h) | -5 | -14 | SIK2(h) | -2 | -24 |
| Fms(h) | -16 | -7 | SIK3(h) | 0 | 3 |
| Fyn(h) | 10 | -1 | SLK(h) | 1 | -1 |
| GAK(h) | 13 | 9 | Snk(h) | -1 | -16 |
| GCK(h) | -11 | -7 | SNRK(h) | -7 | -9 |
| GCN2(h) | -12 | -15 | SRMS(h) | -23 | -15 |
| GRK1(h) | -10 | -14 | SRPK1(h) | -4 | 2 |
| GRK2(h) | 0 | -2 | SRPK2(h) | -3 | -7 |
| GRK3(h) | -17 | -7 | STK16(h) | -10 | 3 |
| GRK4(h) | -6 | -1 | STK25(h) | 14 | 24 |
| GRK5(h) | -11 | -7 | STK32A(h) | -9 | -6 |
| GRK6(h) | -5 | -5 | STK32B(h) | -7 | -6 |
| GRK7(h) | -1 | -4 | STK32C(h) | 2 | 5 |
| GSK3α(h) | -6 | -9 | STK33(h) | -2 | 3 |
| GSK3β(h) | -8 | -2 | STK39(h) | 11 | 11 |
| Haspin(h) | -5 | 0 | Syk(h) | -1 | -16 |
| Hck(h) | 2 | 9 | TAF1L(h) | -12 | -8 |
| Hck(h) activated | 1 | 4 | TAK1(h) | -6 | 27 |
| HIPK1(h) | -2 | -7 | TAO1(h) | -5 | 4 |
| HIPK2(h) | -5 | -3 | TAO2(h) | 3 | 2 |
| HIPK3(h) | -3 | 7 | TAO3(h) | -4 | -6 |
| HIPK4(h) | 1 | 1 | TBK1(h) | 3 | 7 |
| HPK1(h) | -9 | -14 | Tec(h) activated | -6 | 19 |
| HRI(h) | -13 | -7 | TGFBR1(h) | -10 | -10 |
| ICK(h) | -11 | 7 | TGFBR2(h) | -13 | 6 |
| IGF-1R(h) | -13 | -17 | Tie2 (h) | 0 | 12 |
| IGF-1R(h), activated | -2 | -18 | TLK1(h) | -13 | -23 |
| IKKα(h) | -13 | -7 | TLK2(h) | -13 | 8 |
| IKKβ(h) | -5 | -22 | TNIK(h) | -21 | -13 |
| IKKε(h) | 3 | -9 | TRB2(h) | -12 | 4 |
| IR(h) | -1 | -1 | TrkA(h) | -9 | -3 |
| IR(h), activated | -13 | -15 | TrkB(h) | 0 | 4 |
| IRE1(h) | 4 | -2 | TrkC(h) | 1 | 9 |
| IRR(h) | -2 | -1 | TSSK1(h) | 7 | 17 |
| IRAK1(h) | 3 | -8 | TSSK2(h) | -7 | -6 |
| IRAK4(h) | -17 | -11 | TSSK3(h) | -5 | -15 |
| Itk(h) | -3 | -9 | TSSK4(h) | 1 | 4 |
| JAK1(h) | -4 | -1 | TTBK1(h) | -10 | -20 |
| JAK2(h) | -3 | 2 | TTBK2(h) | -9 | 9 |
| JAK3(h) | -6 | -21 | TTK(h) | -10 | -12 |
| JNK1α1(h) | -1 | 8 | Txk(h) | -3 | -3 |
| JNK2α2(h) | -1 | -3 | TYK2(h) | 0 | -14 |
| JNK3(h) | -7 | 1 | ULK1(h) | -5 | -7 |
| KDR(h) | 10 | 24 | ULK2(h) | -8 | 2 |
| LATS1(h) | 9 | 10 | ULK3(h) | -8 | -6 |
| LATS2(h) | -8 | -15 | VRK1(h) | -6 | 3 |
| Lck(h) | -17 | -12 | VRK2(h) | -4 | -11 |
| Lck(h) activated | 3 | -13 | Wee1(h) | -8 | -3 |
| LIMK1(h) | -15 | -21 | Wee1B(h) | -6 | -3 |
| LIMK2(h) | 4 | 5 | WNK1(h) | -16 | 19 |
| LKB1(h) | -3 | 1 | WNK2(h) | -6 | 5 |
| LOK(h) | -5 | 2 | WNK3(h) | 9 | 3 |
| Lyn(h) | -8 | -24 | WNK4(h) | -10 | -4 |
| LRRK2(h) | 16 | 5 | Yes(h) | 0 | 2 |
| LTK(h) | -12 | -4 | ZAK(h) | 0 | -6 |
| MAK(h) | 4 | 5 | ZAP-70(h) | -11 | 6 |
| MAPK1(h) | -11 | -14 | ZIPK(h) | 0 | -9 |
| MAPK2(h) | 3 | 5 | ATM(h) | -12 | -18 |
| MAP4K3(h) | -7 | 2 | ATR/ATRIP(h) | 1 | 2 |
| MAP4K4(h) | -5 | -4 | DNA-PK(h) | 5 | 3 |
| MAP4K5(h) | -1 | -17 | PI3 Kinase (p110β/p85α)(h) | 5 | 4 |
| MAPKAP-K2(h) | -2 | -4 | PI3 Kinase (p120γ)(h) | 1 | 7 |
| MAPKAP-K3(h) | 5 | 2 | PI3 Kinase (p110δ/p85α)(h) | -1 | 27 |
| MATK(h) | -10 | -15 | PI3 Kinase (p110α/p85α)(h) | -2 | 30 |
| MEK1(h) | 10 | -6 | PI3KC2α(h) | 4 | 15 |
| MEK2(h) | 2 | 3 | PI3KC2γ(h) | -3 | -3 |
| MARK1(h) | -8 | -8 | PIP4K2α(h) | -3 | -1 |
| MARK3(h) | 1 | 8 | PIP5K1α(h) | -4 | -1 |
| MARK4(h) | -6 | -2 | PIP5K1γ(h) | -1 | 0 |

**Supplementary Table 5.** 75 CNS CEREP off-target panel screen. Two off-targets H2 and 5-HT5A showed blocking effect > 70% at 1 µM, highlighted yellow.

| Targets | % of inhibition |
| --- | --- |
| KATP Rat Potassium Ion Channel Mass Spectrometry Binding (Antagonist Ligand) Assay, Cerep | - |
| Non-Selective Rat Glycine Ion Channel Strychnine Mass Spectrometry Binding Assay, Cerep | 13.8 |
| Glutamate (Non-Selective) Rat Ion Channel Glycine (Strychnine-Insensitive),Mass Spectrometry Binding Assay, Cerep | - |
| A1 (h) (antagonist radioligand) | - |
| A2A (h) (agonist radioligand) | 28.5 |
| A3 (h) (agonist radioligand) | - |
| alpha1 (non-selective) (antagonist radioligand) | 47.7 |
| alpha2 (non-selective) (antagonist radioligand) | 46.4 |
| beta1 (h) (agonist radioligand) | - |
| beta2 (h) (antagonist radioligand) | - |
| BZD (central) (agonist radioligand) | - |
| B2 (h) (agonist radioligand) | - |
| CB2 (h) (agonist radioligand) | - |
| CB1 (h) (agonist radioligand) | - |
| CCK1 (CCKA) (h) (agonist radioligand) | - |
| CCK2 (CCKB) (h) (agonist radioligand) | - |
| D1 (h) (antagonist radioligand) | 40.5 |
| D2S (h) (agonist radioligand) | 60.5 |
| ETA (h) (agonist radioligand) | - |
| GABA (non-selective) (agonist radioligand) | - |
| AMPA (agonist radioligand) | - |
| xidase (agonist radioligand) | - |
| NMDA (antagonist radioligand) | - |
| H1 (h) (antagonist radioligand) | - |
| H2 (h) (antagonist radioligand) | 90.7 |
| MT1 (ML1A) (h) (agonist radioligand) | 14 |
| M1 (h) (antagonist radioligand) | 60.5 |
| M2 (h) (antagonist radioligand) | 16.5 |
| M3 (h) (antagonist radioligand) | - |
| M4 (h) (antagonist radioligand) | 38.1 |
| M5 (h) (antagonist radioligand) | 11.6 |
| NK1 (h) (agonist radioligand) | - |
| NK2 (h) (agonist radioligand) | - |
| NK3 (h) (antagonist radioligand) | - |
| Y (non-selective) (agonist radioligand) | 12 |
| NTS1 (NT1) (h) (agonist radioligand) | - |
| N neuronal alpha4beta2 (h) (agonist radioligand) | - |
| N muscle-type (h) (antagonist radioligand) | - |
| delta (DOP) (h) (agonist radioligand) | 16.8 |
| kappa (h) (KOP) (agonist radioligand) | 24.4 |
| mu (MOP) (h) (agonist radioligand) | - |
| PPARgamma h) (agonist radioligand) | - |
| PCP (antagonist radioligand) | - |
| EP2 (h) (agonist radioligand) | - |
| EP4 (h) (agonist radioligand) | - |
| IP (PGI2) (h) (agonist radioligand) | 25.3 |
| 5-HT1A (h) (agonist radioligand) | 18.1 |
| 5-HT1B (h) (antagonist radioligand) | 45.3 |
| 5-HT2A (h) (agonist radioligand) | 39.8 |
| 5-HT2B (h) (agonist radioligand) | 42.3 |
| 5-HT2C (h) (antagonist radioligand) | 66.7 |
| 5-HT3 (h) (antagonist radioligand) | - |
| 5-HT5A (h) (agonist radioligand) | 87.5 |
| 5-HT6 (h) (agonist radioligand) | 58.6 |
| 5-HT7 (h) (agonist radioligand) | 53.9 |
| sigma (non-selective) (h) (agonist radioligand) | 20.4 |
| sst (non-selective) (agonist radioligand) | - |
| GR (h) (agonist radioligand) | - |
| PR (h) (agonist radioligand) | - |
| AR(h) (agonist radioligand) | 12.2 |
| BZDp (TSPO) (h) (antagonist radioligand) | 31.4 |
| V1a (h) (agonist radioligand) | - |
| KV channel (antagonist radioligand) | - |
| SKCa channel (antagonist radioligand) | - |
| norepinephrine transporter (h) (antagonist radioligand) | 12.6 |
| dopamine transporter (h) (antagonist radioligand) | - |
| GABA transporter (antagonist radioligand) | - |
| 5-HT transporter (h) (antagonist radioligand) | 18.9 |
| COX1(h) | - |
| COX2(h) | - |
| PDE3A (h) | - |
| PDE4D2 (h) | - |
| Lck kinase (h) | - |
| acetylcholinesterase (h) | 17.3 |
| MAO-A Human monoamine xidase A, Enzymatic assay, Cerep | - |

**Supplementary Table 6.** Calculated half-life (t1/2) and intrinsic clearance of JNJ-TDP43-1 in human, monkey, and rat hepatocytes


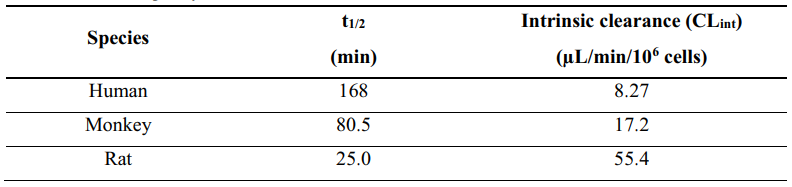


**Supplementary Table 7.** Comparative table showing [¹⁸F]JNJ‑TDP43‑1 and [¹⁸F]ACI‑19626 tracers side by side.

**Supplementary Figures and figure legends**
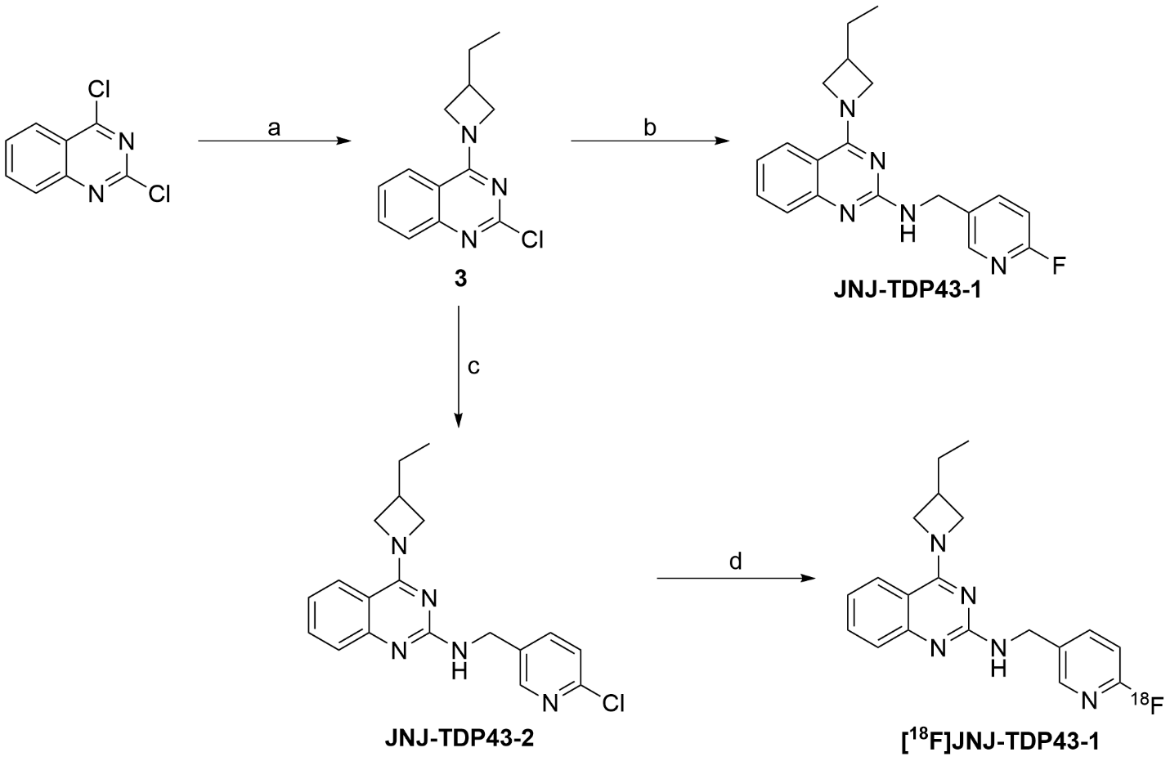


**Supplementary Figure 1**. Synthesis of cold JNJ-TDP43-1 and radio-labeled [^18^F]JNJ-TDP43-1. a) 3-ethylazetidine trifluoroacetate, DIPEA, acetonitrile, 0 °C, 2 h. b) (6-fluoropyridin-3-yl) methenamine, EtOH, mW, 150 °C, 2h. c) (6-chloropyridin-3-yl) methenamine, EtOH, mW, 150 °C, 2h. d) [^18^F]KF, K_2_CO_3_, Kryptofix-222, DMSO, 180 °C, 10 min.

**
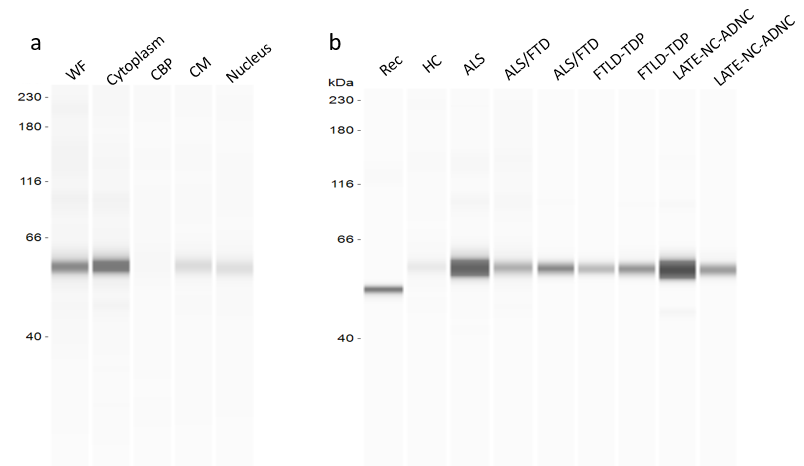
**

**Supplementary Figure 2.** Western blot characterization of TDP-43 pathology in human brain homogenates. a. pTDP-43 level in subcellular fractionations measured by WB. b. WB analysis of p-TDP-43 in normal and disease brain cytoplasm.

**Abbreviates: a.** Fraction labels: WF, whole fraction; CBP, chromatin binding portion; CM, cell membrane fraction. **b.** Lane labels: Rec, recombinant TDP-43 protein; HC, healthy control; ALS, amyotrophic lateral sclerosis; ALS/FTD, amyotrophic lateral sclerosis with frontotemporal dementia; FTLD-TDP, frontotemporal lobar degeneration with TDP-43 pathology; LATE-NC/ADNC, limbic-predominant age-related TDP-43 encephalopathy neuropathologic change/Alzheimer’s disease neuropathologic change.


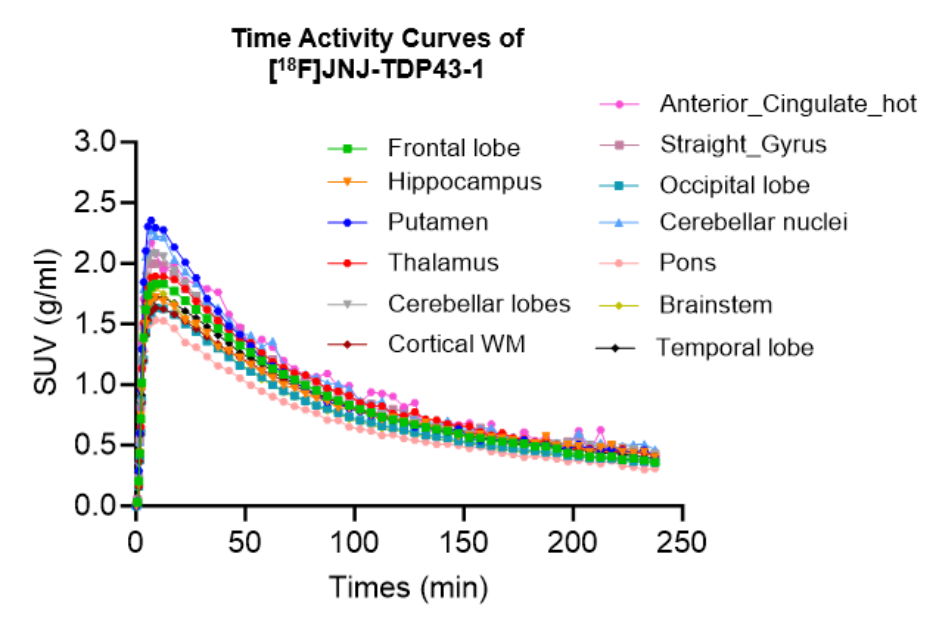


**Supplementary Figure 3.**TACs (SUV, g/mL) from selected brain regions of [^18^F]JNJ-TDP43-1 in rhesus macaque, from 0–240 min post injection.


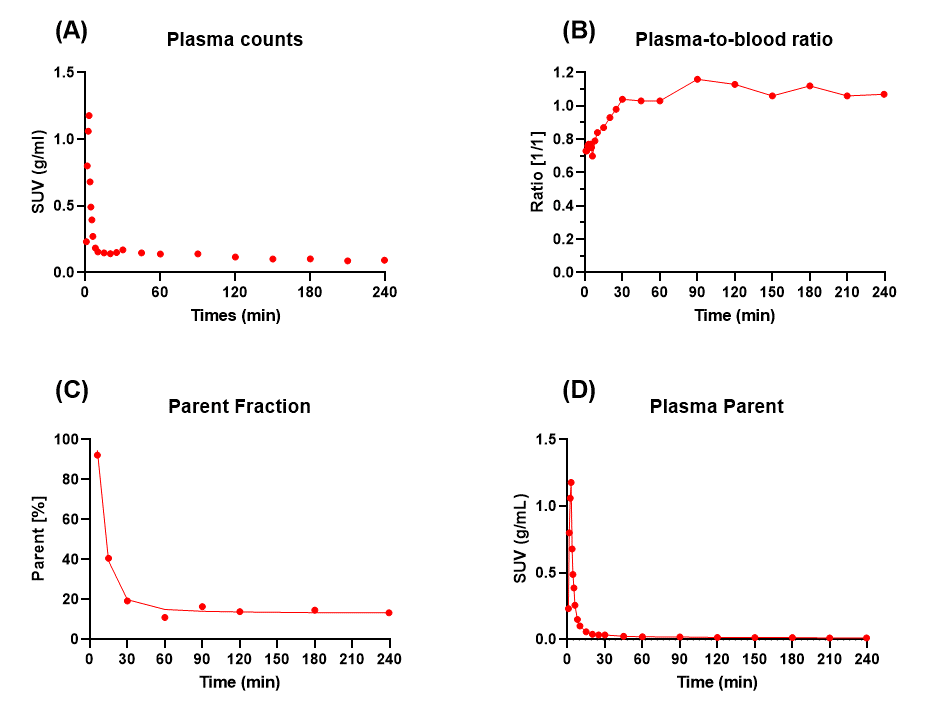


**Supplementary Figure 4.** (A) Plasma radioactivity (expressed as SUV), (B) plasma-to-blood partition, (C) parent fraction (fit with a sigmoid function) and (D) plasma parent fraction of [^18^F]JNJ-TDP43-1 over 0–240 min following IV bolus injection.

**
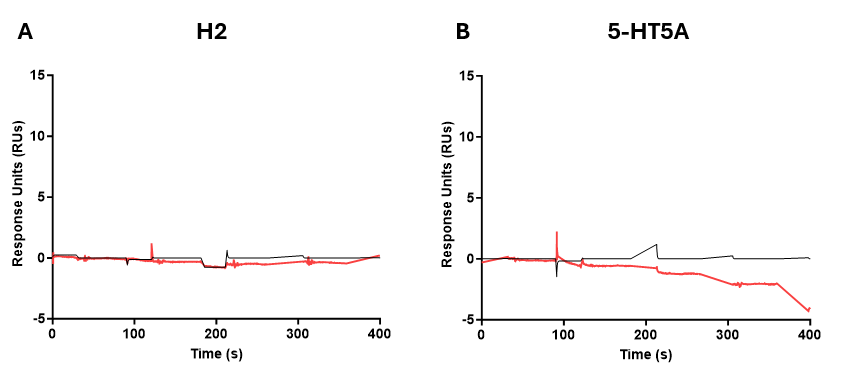
**

**Supplementary Figure 5.** Representative sensorgrams displaying poor binding of JNJ-TDP43-1 to histamine H2 (A) and serotonin 5-HT5A (B) receptors captured through anti-H2 and anti-5-HT5A antibodies in human healthy control (HC) brain homogenate.

[^18^F]JNJ-TDP43-1 Metabolism


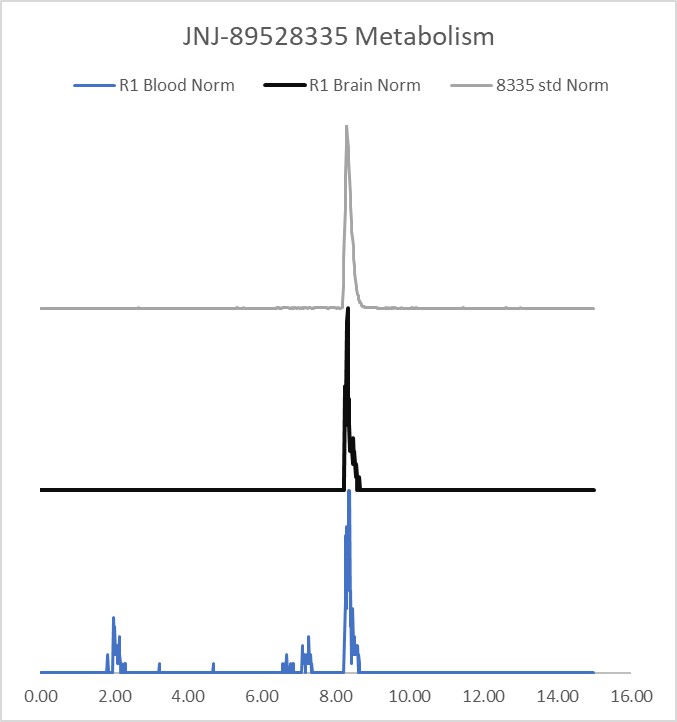


[^18^F]JNJ-TDP43-1 Standard

[^18^F]JNJ-TDP43-1 in Brain

[^18^F]JNJ-TDP43-1 in Blood

**A**

**B**

**C**

**Supplementary Figure 6. HPLC chromatograms of [^18^F]JNJ-TDP43-1 in vivo metabolite profiles.** (A) Radioactive standard of [^18^F]JNJ-TDP43-1. (B) HPLC radio-chromatogram of [^18^F]JNJ-TDP43-1 30 min post-injection in brain. (C) HPLC radio-chromatogram of [^18^F]JNJ-TDP43-1 in blood supernatant 30 min post-injection.
